# Estimating sleep duration: performance of open-source processing of actigraphy compared to in-laboratory polysomnography in the community

**DOI:** 10.1093/sleepadvances/zpad028

**Published:** 2023-07-20

**Authors:** Kelly Sansom, Amy Reynolds, Joanne McVeigh, Diego R Mazzotti, Satvinder S Dhaliwal, Kathleen Maddison, Jennifer Walsh, Bhajan Singh, Peter Eastwood, Nigel McArdle

**Affiliations:** Centre for Sleep Science, School of Human Sciences, University of Western Australia, Perth, WA, Australia; West Australian Sleep Disorders Research Institute, Sir Charles Gairdner Hospital, Perth, WA, Australia; Flinders Health and Medical Research Institute, College of Medicine and Public Health, Flinders University, Adelaide, SA, Australia; Flinders Health and Medical Research Institute, College of Medicine and Public Health, Flinders University, Adelaide, SA, Australia; Curtin School of Allied Health, Faculty of Health Sciences, Curtin University, Perth, WA, Australia; Movement Physiology Laboratory, School of Physiology, University of Witwatersrand, South Africa; Division of Medical Informatics, Department of Internal Medicine, University of Kansas Medical Center, KS, USA; Division of Pulmonary, Critical Care and Sleep Medicine, Department of Internal Medicine, University of Kansas Medical Center, KS, USA; Curtin Health Innovation Research Institute, Faculty of Health Sciences, B305, Curtin University, Bentley, WA, Australia; Office of the Provost, Singapore University of Social Sciences, Clementi Road, Singapore; Duke-NUS Medical School, National University of Singapore, 8 College Road, Singapore; Institute for Research in Molecular Medicine (INFORMM), Universiti Sains Malaysia, Pulau Pinang, Malaysia; Centre for Sleep Science, School of Human Sciences, University of Western Australia, Perth, WA, Australia; West Australian Sleep Disorders Research Institute, Sir Charles Gairdner Hospital, Perth, WA, Australia; Department of Pulmonary Physiology and Sleep Medicine, Sir Charles Gairdner Hospital, Perth, WA, Australia; Centre for Sleep Science, School of Human Sciences, University of Western Australia, Perth, WA, Australia; West Australian Sleep Disorders Research Institute, Sir Charles Gairdner Hospital, Perth, WA, Australia; Department of Pulmonary Physiology and Sleep Medicine, Sir Charles Gairdner Hospital, Perth, WA, Australia; Centre for Sleep Science, School of Human Sciences, University of Western Australia, Perth, WA, Australia; West Australian Sleep Disorders Research Institute, Sir Charles Gairdner Hospital, Perth, WA, Australia; Department of Pulmonary Physiology and Sleep Medicine, Sir Charles Gairdner Hospital, Perth, WA, Australia; Flinders Health and Medical Research Institute, College of Medicine and Public Health, Flinders University, Adelaide, SA, Australia; Centre for Sleep Science, School of Human Sciences, University of Western Australia, Perth, WA, Australia; West Australian Sleep Disorders Research Institute, Sir Charles Gairdner Hospital, Perth, WA, Australia; Department of Pulmonary Physiology and Sleep Medicine, Sir Charles Gairdner Hospital, Perth, WA, Australia

**Keywords:** accelerometry, methodology, triaxial, non-proprietary

## Abstract

Comparisons of actigraphy findings between studies are challenging given differences between brand-specific algorithms. This issue may be minimized by using open-source algorithms. However, the accuracy of actigraphy-derived sleep parameters processed in open-source software needs to be assessed against polysomnography (PSG).

Middle-aged adults from the Raine Study (*n* = 835; F 58%; Age 56.7 ± 5.6 years) completed one night of in-laboratory PSG and concurrent actigraphy (GT3X+ ActiGraph). Actigraphic measures of total sleep time (TST) were analyzed and processed using the open-source R-package *GENEActiv and GENEA data in R (GGIR) with* and without a sleep diary and additionally processed using proprietary software, ActiLife, for comparison. Bias and agreement (intraclass correlation coefficient) between actigraphy and PSG were examined. Common PSG and sleep health variables associated with the discrepancy between actigraphy, and PSG TST were examined using linear regression.

Actigraphy, assessed in GGIR, with and without a sleep diary overestimated PSG TST by (mean ± SD) 31.0 ± 50.0 and 26.4 ± 69.0 minutes, respectively. This overestimation was greater (46.8 ± 50.4 minutes) when actigraphy was analyzed in ActiLife. Agreement between actigraphy and PSG TST was poor (ICC = 0.27–0.44) across all three methods of actigraphy analysis. Longer sleep onset latency and longer wakefulness after sleep onset were associated with overestimation of PSG TST.

Open-source processing of actigraphy in a middle-aged community population, agreed poorly with PSG and, on average, overestimated TST. TST overestimation increased with increasing wakefulness overnight. Processing of actigraphy without a diary in GGIR was comparable to when a sleep diary was used and comparable to actigraphy processed with proprietary algorithms in ActiLife.

**Statement of Significance**

Actigraphy is increasingly used to provide a convenient cost-effective measure of objective sleep duration in adults. However, data validating actigraphy against the reference standard in-laboratory polysomnography are limited by relatively small study samples and the use of brand-specific proprietary actigraphic algorithms. These limitations might be resolved by studying larger well-defined samples and using open-source methods to process actigraphy data. This is the first study to assess the performance of actigraphy, analyzed using an open-source R-package, compared to in-laboratory polysomnography, in a large middle-aged community population.

## Introduction

Polysomnography (PSG) is the reference standard objective measurement of sleep since it can accurately characterize sleep using electroencephalography (EEG) [1]. However, PSG requires specialized expertise, is time-consuming and costly, and due to its intrusiveness can influence an individual’s ability to sleep. This makes PSG impractical for some purposes, particularly when the primary measure of interest is sleep duration over multiple nights in a large group of individuals. Actigraphy devices, typically worn on the wrist, are a more accessible and affordable approach for estimating habitual sleep duration over multiple nights. Actigraphy devices contain built-in accelerometers capable of sensing acceleration and deceleration movements of the wrist on one or more planes (for example, x, y, or z) [2]. The absence of movement is used as a marker of sleep. As a period of motionless wakes, such as reading in bed, might be misclassified as sleep, self-reported lights off and final wake times from sleep diaries are often used to improve the estimation of sleep by actigraphy.

Studies validating actigraphy against PSG in adults have shown generally acceptable agreement, supporting the use of this methodology [3–11]. However, there are a number of limitations to the validation studies. First, actigraphy data have usually been analyzed using proprietary algorithms in brand-specific software, which lack transparency, reproducibility, and consistency as algorithms may differ between actigraphy device types [2]. [12]^,^ This makes it difficult to compare results between studies using different device types [13]. A potential solution to this problem is to use open-source software to analyze actigraphy data, as this provides publicly available code that is easily reproducible and can improve comparability of outcomes between devices and studies [14]. Second, validation studies have mainly included either small numbers of healthy participants [5, 6, 8, 10], or clinical populations referred for sleep-related complaints [3, 7, 11]; there are few data comparing actigraphy to PSG from large, community-based adult populations [4, 9]. It is likely that the performance of actigraphy will vary according to the population studied [5, 15, 16]. Many studies suggest that poor sleep and the presence of sleep disorders may be important factors affecting actigraphy performance [4, 7, 11]. [16]^,^ However, our understanding of the factors that influence the performance of actigraphy in well-defined community samples with open-source analysis is limited.

The present study examined the agreement between actigraphy and PSG in a large community-based study. A recent follow-up of middle-aged participants from the Raine Study who underwent a comprehensive health assessment including health questionnaires, overnight PSG, and multiple nights of actigraphy [17] provided an opportunity to compare actigraphy and PSG in those participants who completed both on the same night. A commonly used open-source package, GGIR (GENEActiv and GENEA data in R), were used [18]. This package calculates sleep and wakeful activity from raw acceleration data acquired from many types of actigraphy devices and has been validated against PSG in a small sample of patients from a sleep clinic population (*n* = 28) [13] and healthy volunteers (*n* = 22) [13, 19]. Sleep detection in GGIR can be guided either by a sleep diary [13] or determined without a sleep diary [19] using the GGIR heuristic called the HDCZA algorithm.

The present study also aimed to compare the performance of GGIR-processed actigraphy to actigraphy processed in the brand-specific software ActiLife against PSG. This is of importance given studies may compare sleep metrics derived from actigraphy processed and analyzed by different brands or software. It is unclear if sleep metrics such as PSG TST are better estimated when actigraphy is processed in GGIR or in ActiLife. Hence, the current study additionally processed the raw acceleration data in ActiLife and used a participant's sleep diary, the Tudor-Locke and Cole-Kripke algorithms to detect sleep.

Therefore, the current study presents comparative data for actigraphy analyzed in GGIR, with and without a sleep diary, and actigraphy data analyzed in ActiLife relative to in-laboratory PSG. Commonly derived actigraphic sleep duration metrics of total sleep time (TST), sleep onset latency (SOL), wake after sleep onset (WASO), and sleep efficiency (SE) were calculated. As habitual sleep duration is a consistent determinant of long-term health outcomes in adults [20–23], in this study TST is the primary outcome of interest. Hence, the main aims were to determine (1) the agreement of actigraphy-derived TST using the open-source R-package GGIR, with and without sleep diary data and using proprietary algorithms in ActiLife software compared to in-laboratory PSG TST, and (2) the variables influencing the performance of open-source actigraphy for estimation of TST. Informed by previous literature a number of potentially influential factors were considered, including PSG characteristics of poor sleep (e.g. increased SOL and WASO) and sleepiness, as well as, the presence and severity of common sleep disorders, obstructive sleep apnea (OSA), and insomnia. It was hypothesized that actigraphy-derived TST would have good agreement with PSG TST in most participants, but have poorer agreement in some subgroups of participants, particularly those with PSG markers of poor sleep and those with common sleep disorders such as OSA.

## Methods

### Study population

Data analyzed in this study were obtained from participants in the Raine study [17]. The Raine study is an ongoing Western Australian pregnancy cohort study that recruited 2900 pregnant women from the community in 1989–1991. The parents studied were designated generation (gen) 1 and their children were designated gen 2. Gen 1 participants were followed up between April 22, 2015 to June 16, 2017, when their children (the gen 2 participants) were approximately 26 years of age (designated gen 1–26 year follow up).

The Raine study design has been described in detail elsewhere [17]. Briefly, the Gen1-26 year follow-up (*n* = 1098) comprised a broad series of assessments including questionnaires, anthropometric measures, and sleep assessments. The sleep assessments included a concurrent actigraphy and PSG night, along with completion of a sleep diary, to guide the actigraphy analysis. Participants were excluded from analyzes if the PSG TST was <2 hours, poor PSG respiratory signal was reported by the sleep scientist, there was no actigraphy file, no sleep diary, actigraphy not simultaneous with PSG, <2 hours of actigraphy TST or missing information on sleep comorbidities including insomnia symptoms and daytime sleepiness symptoms (further information on the number of participants excluded are summarized in the results section). Written and informed consent was obtained from all participants and ethics approval was granted by the University of Western Australia Human Research Ethics Committee (RA/4/7236 *Adult Sleep Study: Prevalence, Phenotype & Genotype of Common Sleep Disorders*).

### Actigraphy

Participants wore an ActiGraph device (GT3X+ ActiGraph LLC, Pensacola, FL; sampling frequency was 30 Hz; idle sleep mode not enabled) on their non-dominant wrist for 8 nights and completed a sleep diary every morning noting the time they turned the lights off and time they woke up in the morning. The first night of actigraphy coincided with the PSG study night. ActiGraph raw data were downloaded to the manufacturer’s software ActiLife (ActiGraph LLC, Pensacola, FL; version 6.13.4). The raw accelerometry data, expressed in gravitational units (m/s^2^), were exported as a CSV file and imported into R-studio, along with sleep diary information, and analyzed using the R-package GGIR (version 2.6.0) [13, 18]. Additionally, the raw actigraphy data were saved as 60-second epoch AGD files in ActiLife and were later used to process and analyze the data with sleep diaries in the ActiLife software.

### GGIR sleep detection

**Figure 1.**
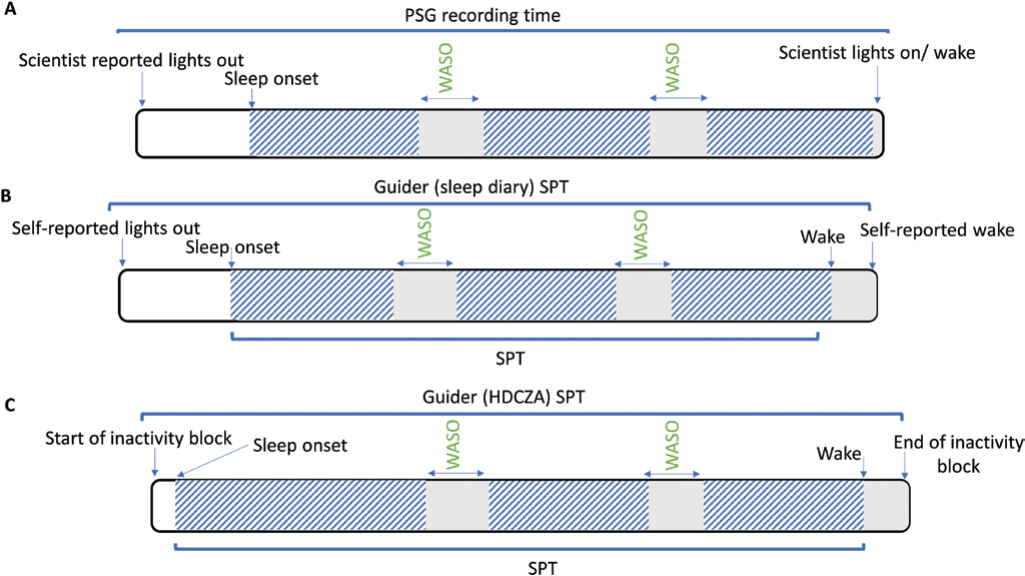
A schematic representation of sleep and wake time definitions when determined by PSG, sleep diary, and HDCZA guider. (A) the diagonal lines represent sleep determined by PSG electroencephalography. (B) The diagonal lines represent sleep determined by sustained inactivity bouts within the sleep diary SPT window. (C) The diagonal lines represent sleep by sustained inactivity bouts within the HDCZA guider SPT window. Abbreviations: PSG, Polysomnography.

The GGIR sleep detection algorithm defined sleep based on periods of inactivity, referred to as sustained inactivity bouts (SIB) [13]. The algorithm classifies periods of time as SIB if the arm angle (*z*-axis) did not change by more than five degrees for a minimum of 5 minutes. The SIB can occur throughout a 24-hour period and may be due to daytime naps, rest, sedentary activity, or the main nightly sleep period time (SPT). To differentiate SIB occurring outside the SPT the GGIR sleep detection algorithm was guided by the sleep diary (lights off and final wake time, GGIR basic sleep diary format) or the GGIR HDCZA algorithm, in separate analyses [13, 19]. Briefly, the HDCZA algorithm estimated the SPT window by identifying the start and end of the longest inactivity period within a 24-hour period (see Van Hees et al. [19] for further details). The time window between reported lights out and waking from the sleep diary or by the HDCZA algorithm were referred to as the guider SPT. The sleep detection algorithm was then used to classify periods of SIB occurring within the guider SPT window as sleep. The first SIB determined by the sleep detection algorithm occurring within the guider SPT window was defined as sleep onset and the end of the last SIB was defined as wake by the sleep detection algorithm. The SPT window was then defined according to the time window between sleep onset and wake derived by the sleep detection algorithm. TST was based on the accumulated SIB within the SPT (see Figure 1 for a schematic representation).

Standard variables from the GGIR output used in the analyses were: sleep onset time, TST (called SleepDurationInSpt), WASO, guider sleep onset time, and SPT (called SptDuration).(cite) The SOL and SE time were not automatically calculated in the standard GGIR output and we calculated separately. The SOL was calculated as difference between guider sleep onset time (sleep diary lights out time) and the sleep detection derived sleep onset time and SE (%) was calculated as TST divided by SPT multiplied by 100. The SOL could not be calculated when the HDCZA algorithm was used as lights-off information was unavailable for these analyses.

### ActiLife analysis

The actigraphy AGD files were imported into ActiLife using the batch sleep processing feature. The Cole-Kripke sleep algorithm [24] was applied to score each 60-second epoch as sleep or wake. The ActiLife-modified Tudor-Locke algorithm [25, 26] was subsequently applied using default settings to detect sleep periods. Briefly, the Tudor-Locke algorithm automatically calculates bedtimes based on the sleep scoring algorithm (e.g. Cole-Kripke) previously applied over the data. The algorithm defines time in bed as 5 consecutive minutes scored as sleep and wake time as 10 consecutive minutes scored as wake after a sleep period. The minimum period between bedtime and wake is 160 minutes and the maximum sleep period length must be no longer than 1440 minutes. After the automatic detection of sleep, the data were cross-checked against the sleep diary. The sleep diary self-reported lights out and wake time was manually entered for each nighttime sleep episode. Automatically scored periods of sleep occurring outside the self-reported sleep diary bedtime and wake times were removed. Standard variables from the ActiLife batch sleep output file were used in the analyses and included: sleep onset, latency, TST, SE, and WASO.

### Polysomnography

One night of in-laboratory PSG (Grael Compumedics, Abbotsford, Victoria, Australia) was conducted at the University of Western Australia Center for Sleep Science. Experienced sleep scientists applied electrodes for measurement of electroencephalography, electrooculography, submental and bilateral leg electromyography, and electrocardiography. Other measurements included oronasal thermistor, nasal airflow, decibel meter for snoring, finger oximetry, thoracic and abdominal inductance plethysmography, and body position monitoring. The PSG was scored blind to the results of actigraphy and using current criteria [27]. The primary comparison variable of interest was TST total time scored as sleep between the recording time (lights off–lights on). We also compared actigraphy to PSG-derived SOL (time of the first epoch of sleep—scientist reported lights out time), WASO (minutes of WASO but before final wake), and SE (TST/recording time). An apnea was defined as a 90% reduction in oronasal thermistor for ≥10 seconds and a hypopnea was defined as ≥30% reduction in nasal airflow lasting ≥10 seconds and accompanied by either an arousal or 3% oxygen desaturation [27]. Presence of OSA was defined as an apnea–hypopnea index (AHI) of ≥5 events/hour and OSA severity was defined as mild (5–15 events/hour), moderate (15–30 events/hour), and severe (≥30 events/hour) [27]. Height and weight measurements were performed using stadiometer and regularly calibrated scales, respectively, by trained research assistants on the evening of the PSG sleep study.”

### Sleep questionnaires

Insomnia symptoms were assessed using the Pittsburgh sleep symptom questionnaire—insomnia [28]. The criteria for having symptoms of chronic insomnia were self-reported trouble falling asleep or difficulty maintaining sleep and feeling sleep was unrefreshing for 3 or more times per week for at least 1-month duration and at least one symptom that impacts daily life (for example, difficulties at work, or social life).

Daytime sleepiness was assessed using the Epworth sleepiness scale (ESS). The ESS is an 8-item self-report questionnaire in which respondents rate their likelihood of dozing in eight different daily circumstances such as watching television or talking to friends [29]. A total score ≥11 is indicative of excessive daytime sleepiness. Questionnaires were completed in the afternoon prior to PSG.

### Sociodemographic assessments

Sociodemographic information was obtained by self-report on sex, age, ethnicity, annual personal income (Australian Dollars; low, <$31 999; medium, $31 200–$64 999; High,>$65 000), education (high school or less, training after school, and university), relationship status (married, widowed, divorced, separated, or de facto), cigarette smoking (current or not), and alcohol intake (abstainer; moderate consumer: 0 < *n* ≤ 4 standard drinks/ day; high consumer: >4 standard drinks/day). These assessments were completed by participants within the month prior to the overnight PSG.

### Statistical analysis

Data analysis were performed in R-Studio (R-Studio Team 2018, Boston MA) and R version 4.0.4 (R Core Teams 2021, Vienna Austria). Descriptive statistics are presented as mean ± SD for continuous variables and count (%) for categorical variables. Comparisons between simultaneous actigraphy and PSG were assessed using paired *t*-tests, the Pearson correlation coefficient, intraclass correlation coefficients (ICC; method: two-way random effects single measures), box and whisker plots, and Bland Altman (BA) plots. Agreement was considered poor if ICC was below 0.5, moderate if values were 0.5–0.75 and good if ICC was above 0.75 [30]. The BA plots present the difference between actigraphy and PSG against PSG sleep duration (reference standard) and show a fitted regression line [31].

The influence of standard PSG variables related to sleep disturbance and common sleep disorders, i.e. OSA, chronic insomnia, and excessive daytime sleepiness (ESS), on the accuracy of the actigraphy estimate of PSG TST were examined using linear regression. The difference between actigraphy and PSG TST was plotted as the dependent variable while sleep-related predictors were plotted as independent variables. A positive beta coefficient indicates actigraphy overestimation of PSG while a negative indicates underestimation and zero indicated no difference. The PSG variables examined were arousal index, WASO, SOL, and SE and OSA severity measures of AHI, arousal index, and 3% oxygen desaturation index. Univariable associations were firstly assessed to determine variables significantly associated with the difference in TST between actigraphy and TST, and then a multivariable model with all variables was constructed to determine significant independent associations. Variables were excluded (a priori *P*-value >0.05) from the multivariable model using backward elimination (R-package “olsrr”) [32], or if multicollinearity existed with another variable. Multicollinearity was assessed using variance inflation factors [33] (values above 10 were considered high). We also performed a sensitivity analysis to compare actigraphy estimates of TST, SOL, WASO, and SE among participants with moderate-severe OSA(AHI ≥15) and no-mild OSA (AHI <15 events/hour).

## Results

### Participant characteristics

**Figure 2.**
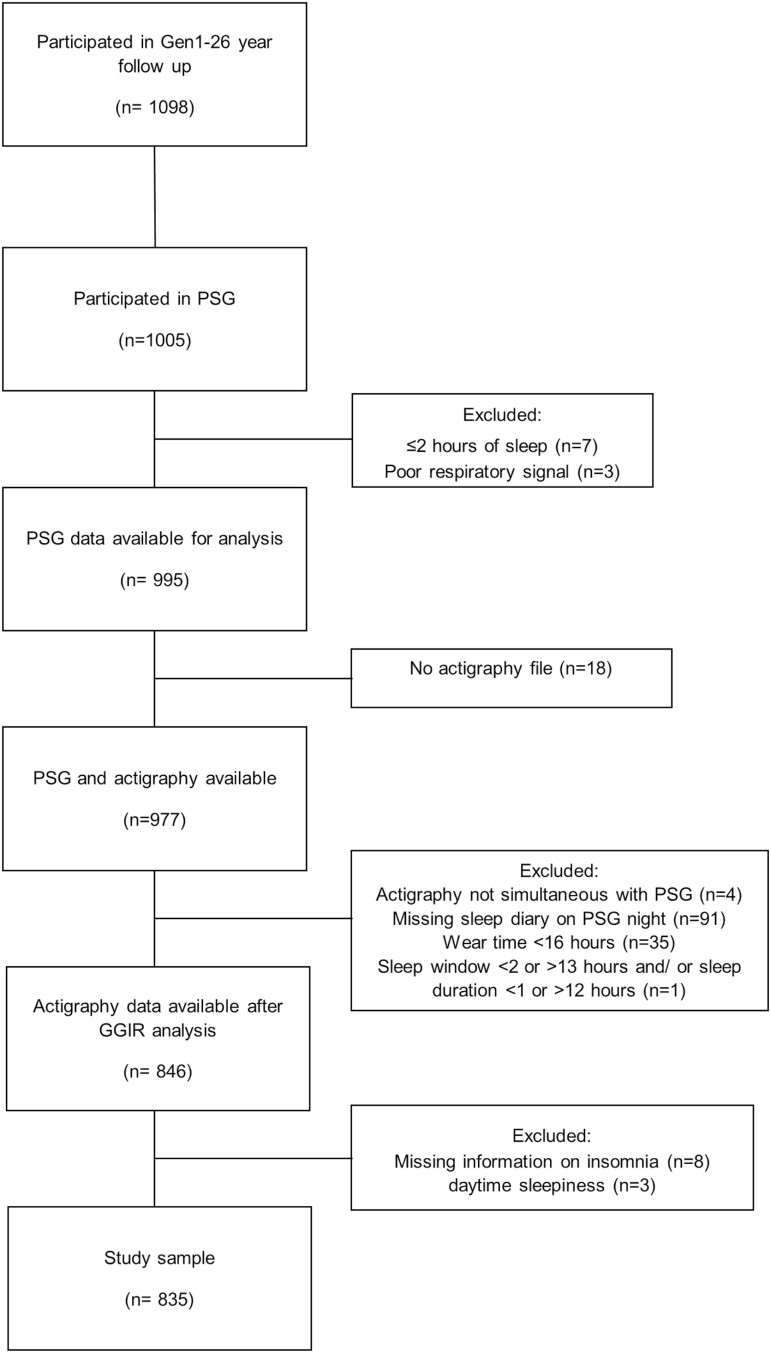
Participant flow diagram.

Figure 2 presents the flow of study participants. Of 1098 gen1–26 year follow-up participants 263 were excluded from this analysis. The most common reasons for exclusion were not undergoing PSG (*n* = 93), missing diary information (*n* = 91), insufficient actigraphy wear time (*n* = 35), or no actigraphy file (*n* = 18). The final sample consisted of 835 participants. With the exception of a slightly increased socioeconomic status the gen1–26 year, follow-up participants are representative of the Australian population on sociodemographic factors and BMI [34]. The sociodemographic characteristics of participants included in this analysis (*n* = 835) were similar to those excluded apart from a greater percentage with higher education attainment, higher income, and nonsmokers in the included sample (*n* = 263; see Supplementary Table S1).

The sample consisted of slightly more women (58%) than men, who were on average 56.7 ± 5.6 years of age, Caucasian (92%), married (72%), achieved an education beyond high school (75%), were in the high-income bracket (44%) and typically overweight (BMI, 28.3 ± 5.6 kg/m^2^). Of the study sample, 8.9% were current smokers and 50% consumed alcohol moderately. Moderate-severe OSA was common (31.8%). Excessive daytime sleepiness and symptoms consistent with chronic insomnia were present in 9.1 and 14% of the sample respectively (see Table 1).

**Table 1.** Sociodemographic and Sleep Characteristics of Participants

Characteristic	*N* = 835^1^
Sex [female]	483 (58%)
Age, years	56.7 ± 5.6
Ethnicity	
* Caucasian*	768 (92%)
* Aboriginal*	4 (0.5%)
* Polynesian*	5 (0.6%)
* Vietnamese*	3 (0.4%)
* Chinese*	23 (2.8%)
* Indian*	26 (3.1%)
* Other*	4 (0.5%)
* Unknown*	2
Relationship status	
* Never Married*	18 (2.2%)
* Married*	582 (72%)
* Widowed*	16 (2.0%)
* Divorced*	108 (13%)
* Separated*	35 (4.3%)
* De facto*	49 (6.1%)
* Unknown*	27
Education level^#^	
* High school or less*	179 (22%)
* Training after school*	302 (38%)
* University*	322 (40%)
* Unknown*	32
Personal income	
* Low*	217 (27%)
* Middle*	229 (29%)
* High*	347 (44%)
* Unknown*	42
Shift Worker	
* Unknown*	715 (88%)
BMI, kg/m^2^	28.3 ± 5.6
Smoker [yes]	71 (8.9%)
* Unknown*	36
Alcohol	
* Alcohol: Abstainer*	255 (33%)
* Medium consumer*	393 (50%)
* High consumer*	134 (17%)
* Unknown*	53
Depression symptoms	121 (15%)
* Unknown*	49
Diabetes [yes]	64 (8.0%)
* Unknown*	32
Insomnia symptoms [present]	119 (14%)
Excessive daytime sleepiness [yes]	76 (9.1%)
PSQI self-reported sleep duration, hours	7.0 ± 1.1
* Unknown*	1
Actigraphy sleep duration on PSG night, hours	6.5 ± 0.8
Polysomnography total sleep time, hours	5.9 ± 1.0
AHI, events/hour	14.6 ± 16.6
OSA severity	
* OSA Free*	248 (30%)
* Mild*	327 (39%)
* Moderate*	151 (18%)
* Severe*	109 (13%)
3% Oxygen desaturation index, desaturations/hour	8.6 (13.6)
Arousal index, events/hour	12.4 ± 14.2
Sleep efficiency, %	78.0 ± 11.5
Sleep onset latency, hours	0.3 ± 0.3
Wake after sleep onset, hours	1.2 ± 0.7

^1^ *n* (%); Mean ± SD.

Abbreviations: BMI, body mass index.

OSA Severity is classified according to AASM guidelines 2012 [27].

^#^The highest education for three individuals was primary school, their demographics are pooled with individuals whose highest education level was high school.

### GGIR actigraphy versus PSG with sleep diary

**Figure 3.**
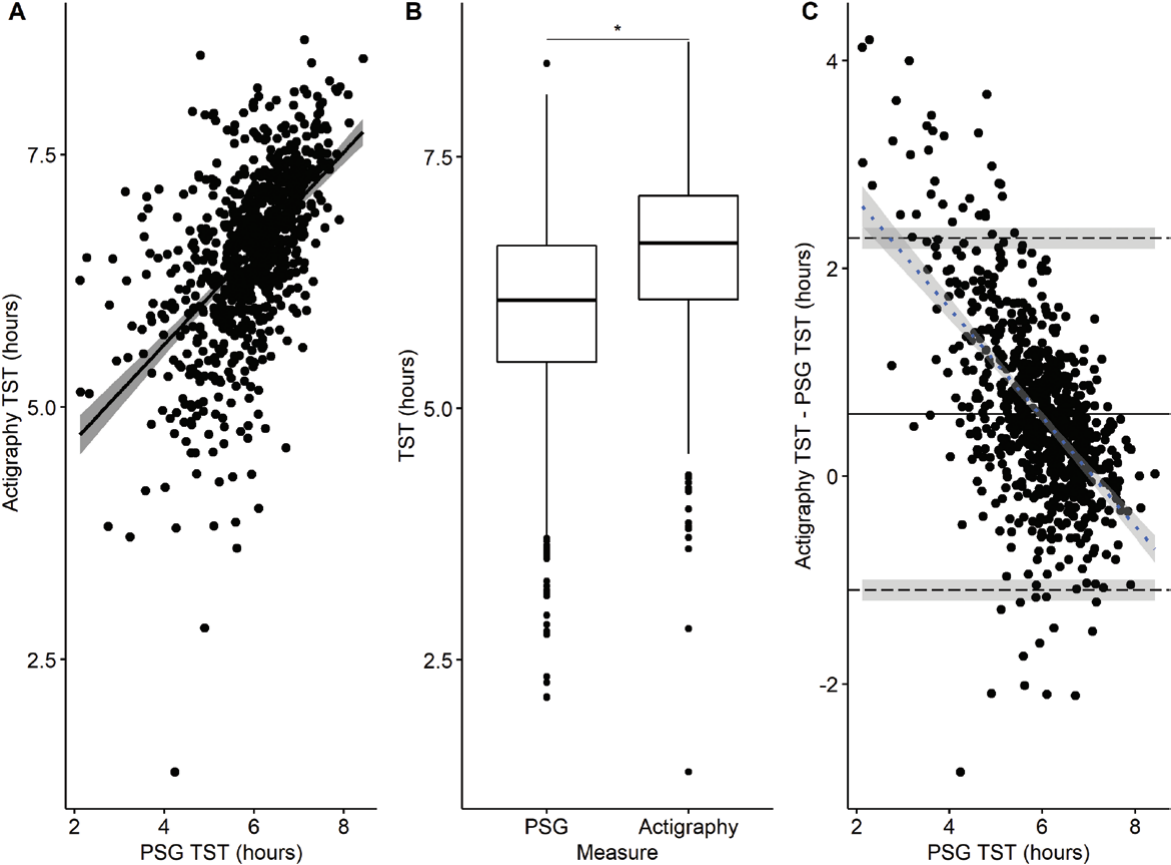
Comparison of GGIR actigraphy with a sleep diary and polysomnography (PSG) measurement of total sleep time (TST) on the same night (*n* = 835). (A) Correlation of actigraphy and PSG TST. (B) Boxplot (median, first and third quartiles, and range) of TST measured from actigraphy and PSG with paired *t*-test. (C) Bland Altman Plot of actigraphy and PSG TST: mean difference (^____^), upper and lower limits of agreement (- - -) with 95% confidence interval (shaded) and regression slope (- - -) with 95% confidence interval (shaded). * *p* < 0.001 for significant differences between actigraphy and PSG.

Figure 3 presents the correlation plot, box plot comparisons, and BA plot of actigraphy analyzed in GGIR compared to PSG TST. A significant correlation (*r* = 0.55, *p* < 0.001) was observed between the two measures. However, the measures of TST were greater with actigraphy than PSG (mean difference = 36 minutes, *p* < 0.001) and the ICC was 0.39 (95% CI: 0.33 to 0.44), indicative of poor agreement. The BA plot (Figure 3C) has lower and upper limits of agreement ranging from −66 to 138 minutes. The regression slope (*r* = −0.58, *p* < 0.001) indicated a proportional bias, with greater overestimation of TST by actigraphy at shorter PSG sleep durations.

The comparison of GGIR actigraphy with a sleep diary and PSG SOL, WASO, and SE are summarized in Table 2. SOL and WASO were on average underestimated, and SE was overestimated by actigraphy. The ICC for actigraphy and PSG SOL, WASO, and SE were poor (ICC: −0.06, 0.29, and 0.13, respectively). The BA and correlation plots are presented in Supplementary Figures S1, S2, and S3.

**Table 2.** Comparison of Actigraphy and PSG Sleep Parameters

Sleep parameter	PSG	Actigraphy	Mean bias	Absolute bias	T statistic	Effect size	*P-*value
	Mean ±SD	Mean ±SD	Mean ±SD	Mean ±SD			
*GGIR: Actigraphy* ***with*** *sleep diary (n = 835)*							
TST, hours	5.95 ± 0.96	6.54 ± 0.84	0.6 ± 0.86	0.78 ± 0.70	19.99	0.69	*<0.001*
SOL, hours	0.29 ± 0.33	0.05 ± 0.17	−0.24 ± 0.35	0.27 ± 0.32	−20.09	−0.7	*<0.001*
WASO, hours	1.25 ± 0.71	0.9 ± 0.61	−0.35 ± 0.74	0.60 ± 0.55	−13.72	−0.47	*<0.001*
SE, %	78.0 ± 11.51	87.21 ± 9.45	9.21 ± 12.1	11.54 ± 9.9	22.00	0.76	*<0.001*
*GGIR: Actigraphy* ***without*** *sleep diary* (*n* = 832)							
TST, hours	5.95 ± 0.95	6.4 ± 1.21	0.44 ± 1.15	0.91 ± 0.84	11.03	0.38	*<0.001*
WASO, hours	1.24 ± 0.7	0.77 ± 0.54	−0.47 ± 0.77	0.66 ± 0.61	−17.46	−0.61	*<0.001*
SE, %	78.09 ± 11.36	88.67 ± 7.48	10.58 ± 11.86	12.18 ± 10.21	25.72	0.89	*<0.001*
*ActiLife: Actigraphy with sleep diary (n = 831)*							
TST, hours	5.95 ± 0.96	6.73 ± 0.76	0.78 ± 0.84	0.87 ± 0.75	27.00	0.94	*<0.001*
SOL, hours	0.29 ± 0.33	0.07 ± 0.08	−0.23 ± 0.34	0.25 ± 0.33	−19.47	−0.68	*<0.001*
WASO, hours	1.25 ± 0.71	0.73 ± 0.5	−0.52 ± 0.71	0.65 ± 0.59	−21.23	−0.74	*<0.001*
SE, %	77.99 ± 11.54	89.54 ± 6.66	11.55 ± 11.02	12.34 ± 10.13	30.21	1.05	*<0.001*

Comparisons were made using paired *t*-tests.

The sample of actigraphy files assessed in GGIR without a sleep diary was reduced by three participants due to TST being calculated as <2 hours, as per exclusion criteria (*n* = 832).

The sample of actigraphy files processed in ActiLife was reduced by four participants as there was an error with their data being formatted into 60-second epochs (sample, *n*= 831).

In the multivariable model (Table 3), PSG-derived SOL, WASO, and AHI explained 70% of the difference observed between actigraphy and PSG TST. Actigraphy overestimated PSG TST by 46.2 (95% CI, 40 to 52) seconds for every minute it took to fall asleep (SOL) and by 47.4 (95% CI, 44 to 50) seconds for every minute spent awake after sleep (WASO). Actigraphy underestimated PSG TST by 2 (95% CI, 0.6 to 3) minutes for each increase in the AHI by 10 events/hour. Symptoms of chronic insomnia and ESS scores did not predict the discrepancy between actigraphy TST with a sleep diary and PSG TST.

**Table 3.** PSG and Sleep Disorder Predictors of the Difference Between GGIR Actigraphy TST With a Sleep Diary and PSG TST (Actigraphy- PSG, Hours)

	Univariable		Multivariable	
Characteristic	Beta (95% CI)^a^	*P*-value	Beta (95% CI)^a^	*P*-value
(Intercept)			−0.58 (−0.65, −0.50)	<0.001
Actigraphy TST (mean centered), hours	0.37 (0.31, 0.44)	<0.001	0.56 (0.52, 0.60)	<0.001
PSG SOL, hours	1.1 (0.91, 1.2)	<0.001	0.77 (0.67, 0.87)	<0.001
PSG WASO, hours	0.71 (0.64, 0.78)	<0.001	0.79 (0.74, 0.84)	<0.001
AHI, per 10 events/hour	−0.03 (−0.06, 0.01)	0.14	−0.03 (−0.05, −0.01)	0.008
Arousal index, events/hour	0 (−0.01, 0.00)	0.2	—	—
Insomnia symptoms	−0.16 (−0.33, 0.01)	0.064	—	—
Daytime sleepiness (ESS)	−0.02 (−0.04, 0.00)	0.011	—	—
PSG 3% Oxygen desaturation index, desaturations/hour	−0.01 (−0.01, 0.00)	0.023	—	—

^a^CI, Confidence Interval.

Multivariable model: R2/R2 adjusted = 0.70/ 0.70.

Variables were not in the multivariable model due to nonsignificance (Insomnia and ESS, *p* > 0.05) or multicollinearity (sleep efficiency, arousal index, and 3% oxygen desaturation index).

### GGIR actigraphy versus PSG without sleep diary

**Figure 4.**
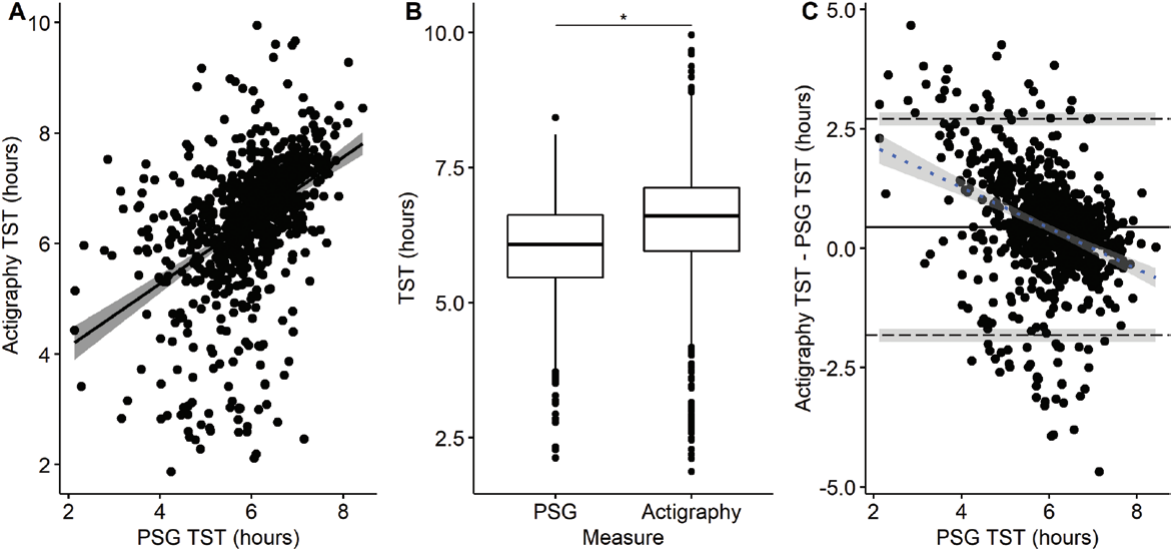
Comparison of GGIR actigraphy without a sleep diary and polysomnography (PSG) measurement of total sleep time (TST) on the same night (*n* = 832). (A) Correlation of actigraphy and PSG TST. (B) Boxplot (median, first and third quartiles, and range) of TST measured from actigraphy and PSG with paired *t*-test. (C) Bland Altman Plot of actigraphy and PSG TST: mean difference (^____^), upper and lower limits of agreement (- - -) with 95% confidence interval (shaded) and regression slope (- - -) with 95% confidence interval (shaded). * *p* < 0.001 for significant differences between actigraphy and PSG.

Figure 4 presents the correlation plot, box plot comparisons, and BA plot of actigraphy analyzed in GGIR without a diary compared to PSG TST. A significant correlation (*r* = 0.45, *p* < 0.001) was observed between the two measures. However, the measures of TST were greater with actigraphy than PSG (mean difference = 26.4 minutes, *p* < 0.001) and the ICC was 0.38 (95% CI: 0.32 to 0.44), indicative of poor agreement. The BA plot (Figure 4C) has lower and upper limits of agreement ranging from −109 to 162 minutes. The regression slope (*r* = −0.35, *p* < 0.001) indicated a proportional bias, with greater overestimation of TST by actigraphy without a diary at shorter PSG sleep durations.

The comparison of PSG and actigraphy WASO and SE are summarized in Table 2. WASO was on average underestimated, and SE was overestimated by actigraphy. The ICC for PSG and actigraphy WASO and SE were poor (ICC: 0.08, 0.05, respectively). The BA and correlation plots are presented in Supplementary Figures S4 and S5.

In the multivariable model (Table 4), PSG-derived SOL and WASO explained 78% of the difference observed between actigraphy-derived without a sleep diary and PSG TST. Actigraphy overestimated PSG TST by 47.4 (95% CI, 40.8 to 54.6) seconds for every minute it took to fall asleep (SOL) and by 48.6 (95% CI, 45.6 to 52.2) seconds for every minute spent awake after sleep (WASO). Symptoms of chronic insomnia, daytime sleepiness and AHI did not predict the discrepancy between the two measures.

**Table 4.** PSG and Sleep Disorder Predictors of the Difference Between GGIR Actigraphy TST Without a Sleep Diary and PSG TST (HDCZA Actigraphy-PSG, Hours)

	Univariable		Multivariable	
Characteristic	Beta (95% CI)^a^	*P*-value	Beta (95% CI)^a^	*P*-value
(Intercept)			−0.8 (−0.88, −0.72)	<0.001
Actigraphy TST (mean-centered), hours	0.64 (0.60, 0.69)	<0.001	0.56 (0.52, 0.60)	<0.001
PSG SOL, hours	0.89 (0.66,1.1)	<0.001	0.79 (0.68, 0.91)	<0.001
PSG WASO, hours	0.56 (0.45, 0.66)	<0.001	0.81 (0.76, 0.87)	<0.001
AHI, per 10 events/hour	−0.08 (−0.12, −0.03)	0.001	—	—
Arousal index, events/hour	−0.01 (−0.01, 0.00)	0.007	—	—
Insomnia symptoms	−0.34 (−0.57, −0.12)	0.003	—	—
Daytime sleepiness (ESS)	−0.03 (−0.05, −0.01)	0.005	—	—
PSG 3% Oxygen desaturation index, desaturations/hour	−0.01 (−0.02, 0.00)	<0.001	—	—

^a^CI, Confidence Interval.

Multivariable model: R2/R2 adjusted = 0.78/ 0.78.

Variables were not in the multivariable model due to nonsignificance (AHI, insomnia, *p* > 0.05) or multicollinearity (sleep efficiency, arousal index, and 3% oxygen desaturation index).

### ActiLife actigraphy versus PSG with sleep diary

**Figure 5.**
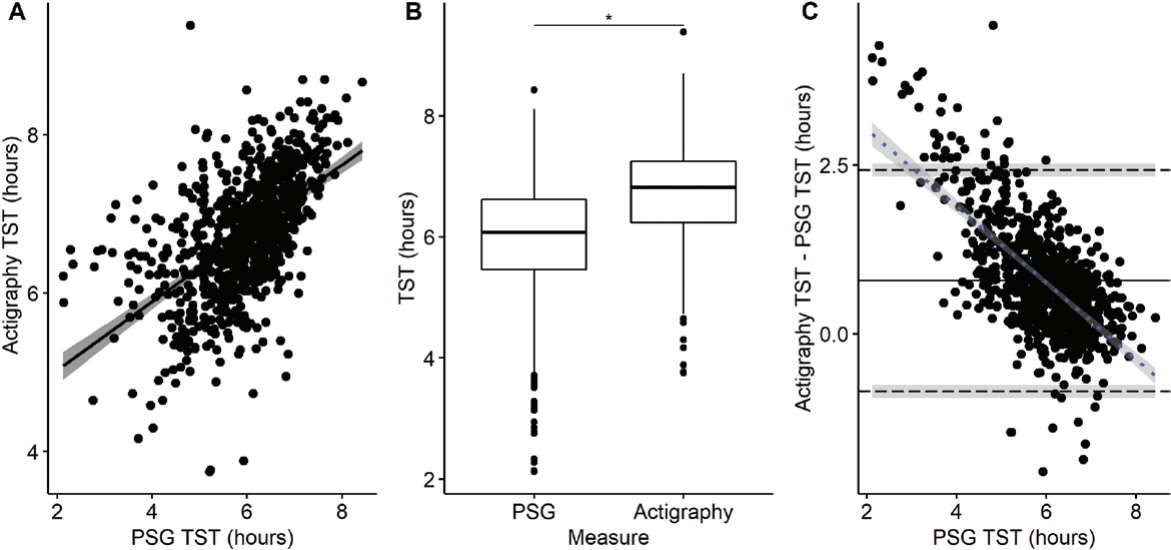
Comparison of actigraphy processed in ActiLife with a sleep diary and polysomnography (PSG) measurement of total sleep time (TST) on the same night (*n* = 831). (A) Correlation of actigraphy and PSG TST. (B) Boxplot (median, first and third quartiles, and range) of TST measured from actigraphy and PSG with paired *t*-test. (C) Bland Altman Plot of actigraphy and PSG TST: mean difference (^____^), upper and lower limits of agreement (- - -) with 95% confidence interval (shaded) and regression slope (- - -) with 95% confidence interval (shaded). * *p* < 0.001 for significant differences between actigraphy and PSG.

On average actigraphy analyzed in ActiLife with a sleep diary overestimated PSG TST by 46.8 minutes (Table 2). Figure 5 presents the correlation plot, box plot comparisons, and BA plot of actigraphy compared to PSG TST. There was a significant correlation between two measures (*r* = 0.55, *p* < 0.001) but agreement was poor (ICC, 95% CI: 0.27, 0.21 to 0.33). The BA plot (Figure 5C) had lower and upper limits of agreement ranging from −51 to 146 minutes. The regression slope (*r* = −0.65, *p* < 0.001) indicated a proportional bias, with greater overestimation of TST by actigraphy at shorter PSG sleep durations.

The comparison of PSG and actigraphy WASO and SE are summarized in Table 2. The SOL and WASO were on average underestimated, and SE was overestimated by actigraphy. The agreement between PSG and actigraphy, SOL, WASO, and SE was poor (ICC: −0.17, 0.14, −0.04, respectively). The BA and correlation plots are presented in Supplementary Figures S6, S7, and S8.

In the multivariable model (Table 5), PSG-derived SOL and WASO explained 73% of the difference observed between actigraphy (processed in ActiLife) and PSG TST. Actigraphy overestimated PSG TST by 48.6 (95% CI, 43.2 to 54.0) seconds for every minute it took to fall asleep (SOL) and by 47.4 (95% CI, 45.0 to 50.4) seconds for every minute spent awake after sleep (WASO). Symptoms of chronic insomnia, daytime sleepiness and AHI did not predict the discrepancy between the two measures.

**Table 5.** PSG and Sleep Disorder Predictors of the Difference Between ActiLife Actigraphy TST With a Sleep Diary and PSG TST (Actigraphy-PSG, Hours)

	Univariable		Multivariable	
Characteristic	Beta (95% CI)^a^	*P*-value	Beta (95% CI)^a^	*P*-value
(Intercept)			−0.45 (−0.51, −0.38)	<0.001
Actigraphy TST (mean-centered), hours	0.3 (0.23, 0.37)	<0.001	0.57 (0.43, 0.51)	<0.001
PSG SOL, hours	1.2 (1.0, 1.3)	<0.001	0.81 (0.72, 0.90)	<0.001
PSG WASO, hours	0.8 (0.74, 0.86)	<0.001	0.79 (0.75,0.84)	<0.001
AHI, per 10 events/hour	0.06 (0.02, 0.09)	0.002	—	—
Arousal index, events/hour	0.01 (0.00, 0.01)	<0.001	—	—
Insomnia symptoms	−0.02 (−0.18, 0.14)	0.8	—	—
Daytime sleepiness (ESS)	−0.02 (−0.03, 0.00)	0.045	—	—
PSG 3% Oxygen desaturation index, desaturations/hour	0 (0.00, 0.01)	0.02	—	—

^a^CI, Confidence Interval.

Multivariable model: R2/R2 adjusted = 0.726/ 0.725.

Variables were not in the multivariable model due to nonsignificance (AHI, insomnia, *p* > 0.05) or multicollinearity (sleep efficiency, arousal index, and 3% oxygen desaturation index).

## Discussion

This study reports for the first time, the performance of open-source actigraphy methods when compared to the same night in-laboratory PSG in a large community sample of middle-aged adults. On average, actigraphy analyzed using the open-source package GGIR overestimated PSG TST by 36 and 26.4 minutes when analyzed with and without a diary, respectively. The overestimation of PSG TST by actigraphy was even greater, 46.8 minutes, when analyzed in proprietary software ActiLife with a sleep diary. While correlation between PSG and actigraphy TST was moderate across the different methods, there was overall poor agreement between actigraphy and PSG-derived TST. The agreement was proportionally worse as indicators of wakefulness observed on PSG increased.

### TST: actigraphy versus PSG

Actigraphy data analyzed in GGIR using a sleep diary as a guider overestimated TST compared to PSG TST, and this was less so when HDCZA algorithm was used. However, when the HDCZA guider was used the BA plots for TST had wider limits of agreement relative to the sleep diary guider and therefore may be less suitable for individual-level data. While the difference in overestimation of TST was 10 minutes greater by the sleep diary this difference may not be of clinical significance but instead highlights the similarity of the two approaches. Overall, these findings suggest that actigraphy without a diary seems to offer advantages for larger epidemiological studies as the HDCZA algorithm performed similarly to the sleep diary and is likely associated with a lower participant impacts and cost. These findings should be considered in the design of future large epidemiological protocols. Indeed, this approach has proved useful for large cohort studies without a sleep diary including the UK Biobank [35] and the US National Health and Nutrition Examination Survey [36]. Although one notable limitation to the use of the HDCZA guider is the lack of information about SOL, which requires “lights out” time according to recommendations for assessment of insomnia [37].

Actigraphy data analyzed using GGIR with a sleep diary has only previously been validated against PSG in one study. van Hees et al. [13] compared the agreement of actigraphy (GENEActiv, Activinsights Ltd, Kimbolton UK; 85.7Hz sampling frequency) to PSG when worn on the non-dominant wrist during one night of in-laboratory PSG in 28 sleep clinic patients (age, 44.9 ± 14.9 years). van Hees et al. [13] found actigraphy overestimated PSG TST by 30 minutes on average. The current study found comparable results but extends these findings to a large middle-aged community population. The current study uses participant-reported lights-out to delimit the start of attempt to sleep for the actigraphy analysis, by contrast, van Hees et al. [13] used the sleep scientist-documented lights-out time. The use of participant-reported “lights out” is an attempt to simulate as much as possible the usual practice where patient report occurs in the home; however, it is recognized that the laboratory environment may not replicate sleep patterns in the home setting.

Actigraphy data analyzed in GGIR using the HDCZA guider has previously been validated against PSG in one study that included a middle-aged sleep clinic cohort (*n* = 28) and a small number of young healthy volunteers (*n* = 22) [19]. In sleep clinic patients, the actigraphy estimate for TST using the GENEActiv (Activinsights Ltd, Kimbolton, UK) device on both wrists was significantly higher than PSG by 30 minutes (*p* = 0.04) for the left wrist but nonsignificantly longer by 18 minutes (*p* = 0.23) for the right wrist (handedness data were not available). In the healthy volunteers, actigraphy using Axivity (Axivity Ltd, Hoults Yard, UK) devices on the non-dominant wrist was more accurate than in the clinical sample; TST by actigraphy was not significantly lower than PSG, on average. The present study found results comparable to the sleep clinic patients, perhaps related to the high prevalence of sleep disorders in our middle-aged community sample, but extends this to a large middle-aged community population.

The overestimation of PSG TST was greatest when actigraphy was analyzed in ActiLife. These findings suggest that actigraphy analyzed in GGIR with or without a sleep diary performs better at estimating PSG TST relative to actigraphy data processed in ActiLife with the Cole-Kripke sleep algorithm. This difference is likely attributed to the fundamental differences in the GGIR sleep scoring algorithm and the Cole-Kripke sleep scoring algorithm applied in ActiLife. The GGIR algorithm scores sleep based on changes in arm angle (accelerometer derived, z angle) whereas the Cole-Kripke algorithm scores sleep based on magnitude of acceleration [13]. It is possible that the novel sleep scoring algorithm employed by GGIR has greater sensitivity for scoring periods of quiet wakefulness during an episode of sleep relative to the Cole-Kripke algorithm.

The overall agreement of actigraphy, assessed with both GGIR and ActiLife, with PSG, was poor. There are a number of validation studies of actigraphy against PSG that found good agreement between these measures [3, 7, 8, 10, 38]; however, those studies appear to have evaluated small, selected populations, such as healthy adults or sleep clinic populations. There are only two prior large community-based studies, that we are aware of, comparing actigraphy (proprietary algorithms) with PSG, and they reported poor to moderate [4], or fair [9] agreement. Hence, it appears that actigraphy performs poorly when evaluated in unselected samples, such as large community settings perhaps related to the degree of diversity in sleep quality and quantity in those settings. Furthermore, underlying comorbidities may also influence the performance of actigraphy in certain subgroups. For example, individuals with conditions that might impact motor activity may have greater discrepancies between PSG and actigraphy estimates [16]. Future research is needed to evaluate the relative performance of actigraphy in different subgroups.

The poor agreement between actigraphy and PSG presumably relates to differences in how each tool measures sleep. Actigraphy sleep is based on arm movements while PSG stages sleep more accurately according to cortical EEG. Therefore, moments of quiet wakefulness are likely to be misclassified by actigraphy as sleep, thus resulting in an overestimation of sleep time. However, currently, the limitations of actigraphy may be outweighed by its low-cost, low participant impact, and ability to collect objective data over many days. Furthermore, sleep quality in the home setting may be greater than in the sleep laboratory and thus may result in improved performance of actigraphy.

### Predictors of actigraphy accuracy for TST

#### Sleep metrics.

Our finding that PSG SOL and WASO were independent predictors of performance of actigraphy is in keeping with epoch-level data from other studies. In particular, previous epoch-level comparisons of actigraphy and PSG demonstrated that both the Cole-Kripke and Sadeh algorithms have high sensitivity (91%–96%) for detecting sleep but low specificity (34%–47%), i.e. poor detection of wake [8, 10]. Hence, our findings are in line with previous research which has shown that actigraphy has low sensitivity to wakefulness [10, 13], and this is thought to occur because people may be relatively motionless as they are attempting to initiate or resume sleep.

### Sleep disorders

Increasing severity of OSA, as assessed by AHI, was associated with underestimation of PSG TST by actigraphy when processed in GGIR with a sleep diary. Consistent with this, in stratified analysis by moderate-severe OSA (AHI ≥15 events/hour) versus no-mild OSA (AHI <15 events/hour) we found that TST was overestimated less among participants with at least moderate OSA relative to lesser severity or no OSA (Supplementary Table S2 and S3). Similar findings were reported in a community population of elderly men [4] and in a clinical population with OSA [11]. Such findings may relate to OSA-associated cortical arousal (sleep fragmentation) and subsequent body movements. Furthermore, such arousals may be associated with relatively brief awakenings which, if they last <15 seconds, are scored as sleep during a 30-second PSG epoch [27]. When actigraphy data were analyzed in GGIR without a sleep diary and in ActiLife with a diary the AHI was not a significant predictor of the difference between actigraphy and PSG, but the reason for this difference, compared to the use with a sleep diary, is unclear. It is possible that, in some people, the use of GGIR without a diary the sleep period incorrectly includes some of the PSG SOL period. This misclassification of wake as sleep, but with no obstructive respiratory events present, would have the effect of weakening the association of AHI with wake/sleep misclassification due to these events and therefore with the difference between actigraphy and PSG TST. Presumably, when using ActiLife, AHI is not a predictor of this difference because the ActiLife software is less sensitive to arousal-related movements during sleep from obstructive respiratory events, compared to GGIR, and so less often misclassifies them as wake.

Excessive daytime sleepiness, a common symptom of OSA, was associated with actigraphy performance in univariable but not in multivariable analysis. The univariable association is consistent with those of Blackwell et al [4] who reported stronger agreement between actigraphy and PSG TST in individuals with excessive daytime sleepiness. However, the effect of ESS on actigraphy performance is not likely to be an independent determinant given that it was not significant in multivariable analysis when other variables including SOL, WASO, SE, and AHI were in the model.

The presence of significant insomnia symptoms was not associated with worse performance by actigraphy. However, objective measures commonly associated with insomnia [39], such as increased SOL and WASO, were strong predictors in the multivariable model. Therefore, these variables may account for the lack of an independent association between symptom-based insomnia diagnosis and performance of actigraphy in the multivariable model.

### Strengths and limitations

This is the first study to assess the performance of actigraphy when processed and analyzed using the open-source R-package GGIR and proprietary software ActiLife, against PSG in a large community population of middle-aged adults. An important strength of this study is the concurrent comparison of actigraphy with the gold standard in-laboratory PSG. However, findings may differ from comparisons done in the home setting in which a “first night” effect and associated sleep disruption are less likely. An advantage of in-laboratory PSG is that signal quality can be optimized, if necessary, by the sleep scientist during the night relative to the home PSG. The study population included similar numbers of males and females, making this study generalizable to both. However, as this is a middle-aged, predominantly Caucasian, community sample the findings may not be generalizable to children, younger or elderly adults, and non-Caucasian populations. This study did not assess epoch-by-epoch comparisons between PSG and actigraphy due to a limitation in resources. However, the aim was to assess whole night measures (commonly used in clinical practice) and not discrete events, similar to previous validation studies [3, 40]. The comparability of ActiGraph data analyzed in GGIR to actigraphy data collected from other devices such as GENEActiv may result in slightly different estimations. Possible inter-device differences may include hardware variabilities such as micromechanical sensors used, dynamic ranges, reference voltage, and analog-to-digital conversion [41–43]. Furthermore, the ActiGraph devices may have on-board processing of the raw data, but that information is proprietary [44]. However, studies have shown that when different devices are used in GGIR the agreement in outcomes are very high [41–43]. Therefore, these findings may be applicable to other studies using different devices in GGIR.

## Conclusion

The present study shows that in a large middle-aged community population, actigraphy analyzed in GGIR with or without a sleep diary moderately overestimates PSG TST. The performance of actigraphy using open-source software against PSG appears to be broadly similar to that of actigraphy using a common proprietary algorithm. Our findings also suggest that in a community setting, there is overall poor agreement between actigraphy and PSG. Furthermore, studies comparing open-source actigraphy to established algorithms may further define the relative performance of open-source methods compared to other algorithms.

## Supplementary Material

zpad028_suppl_Supplementary_Material

## Data Availability

We are willing to share data from this study, according to current Raine study data-sharing rules. The Raine Study holds a rich and detailed collection of data gathered over 30 years for the purpose of health and well-being research. The informed consent provided by each participant does not permit individual-level data to be made available in the public domain (i.e. a public data repository). However, de-identified analytic data sets are available to all researchers for original research or auditing of published findings. All data access is managed through established Raine Study procedures which require data handlers to agree to a code of conduct, outlined in the Raine Study Researcher Engagement Policy, that includes safeguards to protect the identity of participants. Details of the data access processes, and code of conduct are available on the Raine Study website (www.rainestudy.org.au).
