# Supplementary material for: Estimating sleep duration: performance of open-source processing of actigraphy compared to in-laboratory polysomnography in the community
Source: Sleep Adv. 2023 Jul 20;4(1):zpad028. doi: 10.1093/sleepadvances/zpad028 (PMC10362889; doi:10.1093/sleepadvances/zpad028)
Supplement: zpad028_suppl_Supplementary_Material [file zpad028_suppl_supplementary_material.docx]

**Supplementary material**

**Title: Estimating sleep duration: performance of open-source processing of actigraphy compared to in-laboratory polysomnography in the community**

Authors: Kelly Sansom^1,2,3^, Amy Reynolds^3^, Joanne McVeigh^4,5^, Diego Mazzotti^6,7^, Satvinder S. Dhaliwal^8-11^, Kathleen Maddison^1,2,12^, Jennifer Walsh^1,2,12^, Bhajan Singh^1,2,12^, Peter Eastwood^3^, Nigel McArdle^1,2,12^

1. Centre for Sleep Science, School of Human Sciences, The University of Western Australia, Perth, Western Australia, Australia
2. West Australian Sleep Disorders Research Institute, Sir Charles Gairdner, Perth, Western Australia, Australia.
3. Flinders Health and Medical Research Institute, College of Medicine and Public Health, Flinders University, Adelaide, Australia
4. Curtin School of Allied Health, Faculty of Health Sciences, Curtin University, Perth, Western Australia
5. Movement Physiology Laboratory, School of Physiology, University of Witwatersrand, South Africa
6. Division of Medical Informatics, Department of Internal Medicine, University of Kansas Medical Center, Kansas, USA
7. Division of Pulmonary, Critical Care and Sleep Medicine, Department of Internal Medicine, University of Kansas Medical Center, Kansas, USA
8. Curtin Health Innovation Research Institute, Faculty of Health Sciences, B305, Curtin University, Bentley, Western Australia, Australia 6102
9. Singapore University of Social Sciences, 463 Clementi Road, Singapore 599494
10. Duke-NUS Medical School, National University of Singapore, 8 College Rd, Singapore 169857
11. Institute for Research in Molecular Medicine (INFORMM), Universiti Sains Malaysia, 11800 Minden, Pulau Pinang, Malaysia
12. Department of Pulmonary Physiology & Sleep Medicine, Sir Charles Gairdner Hospital, Perth, Western Australia, Australia

Corresponding author: Associate Professor Nigel McArdle

Email: Nigel.McArdle@health.wa.gov.au

**GGIR methods**

The present study utilised data from the Raine Gen1-26-year follow-up which included one night of actigraphy in the sleep-laboratory during polysomnography and seven days of at home actigraphy with a corresponding sleep diary (including time in bed, lights out time, wake time and time of out bedtime). The PSG night occurred on the first night of the study protocol. The actigraphy data was processed in GGIR (version 2.6-0) twice – once with a sleep diary and without a sleep diary. Please see details of analyses steps for each of these below and the provided GGIR syntax.

**Actigraphy processing and cleaning with a basic sleep diary (in-laboratory night) in GGIR**

Self-reported lights out time and wake time from the participants sleep diary was used to guide the actigraphy analysis using the GGIR basic sleep diary format. Individuals with missing diary entries for the PSG night were excluded from the analyses. As per the GGIR user instructions a visual representation of the sleep diary and accelerometer data that differed by more than four hours was applied using the feature “do.visual” and “crit.error” in GGIR part 4.^1^ These diary entries were inspected, and entries were deleted if entries appeared incorrect. In the GGIR code we excluded days with a wear time of <16 hours of wear time from noon to noon.^1^ We analysed the QC/part4_nightsummary_sleep_full.csv file ( see GGIR vignette for further detail of output files, [Accelerometer data processing with GGIR (r-project.org)](https://cran.r-project.org/web/packages/GGIR/vignettes/GGIR.html)). Please note we refer to variables used from this file in italics. We filtered the file by nights that corresponded to the PSG study date and matched with sleep diary. The *sleepparam* column was filtered for T5A5 as sustained inactivity was defined according to a 5-minute window.^1^ We included nights that had an available sleep diary (*cleaningcode* = 0) and excluded nights with a cleaning code of greater than one. The *cleaningcode* one was excluded since this indicates no sleep diary was present and code two indicated non-wear time >16 hours from noon to noon. We further excluded nights with a sleep window of less than two hours or >13 hours or sleep duration in sleep time window of <1 hour or >12 hours as these may be erroneous, as done in a previous paper.^2^ After completing this data cleaning, we had a sample size of n=835.

**Data processing and cleaning without a diary (in-laboratory night)**

*Heuristic HDCZA algorithm*

The Heuristic algorithm estimates the guider sleep period time window based on the distribution of change in the z-angle axis per a 5 minute window.^18^ The lower 10th percentile of values from the z-angle distribution multiplied by 15 is then used as a critical threshold to define periods of time with the least postural changes and movements, ie., inactivity. The algorithm then records blocks of time as inactive if the z-angle axis is below the critical threshold and longer than 30 minutes in duration. Blocks of time recorded as inactive that are <60 minutes apart are combined to form the same block of inactivity. The start and end time of the longest inactivity block between noon and noon are used to define the guider SPT.

Using actigraphy data from the 835 participants used in the previous sleep diary analyses we repeated the processing in GGIR without a sleep diary using the GGIR heuristic HDCZA algorithm. Sleep onset could not be calculated when the Heuristic HDCZA algorithm was used as information on time in bed or lights out would be unavailable without a sleep diary. The same data cleaning process was used except we did not need to clean the sleep diary and the cleaning code one was included. Three individuals were removed from the Heuristic analyses due to insufficient TST (<2 hours).

**GGIR code**

library(GGIR)

#input
f0 = 1
f1=c()
mode= c(1,2,3,4,5)
datadir = "file path"
outputdir = "file path"


g.shell.GGIR(
 mode=mode,
 datadir= datadir,
 outputdir = outputdir,
 f0=f0,
 f1=f1,
 overwrite= FALSE,
 do.imp = TRUE,
 idloc=2,

 #=====================
 # Part 2
 #=====================
 strategy = 1,
 hrs.del.start = 0, hrs.del.end = 0,
 maxdur = 9,
 includedaycrit = 16, # minimum of 16 hours a day of recording
 qwindow=c(0,24), # all variables will be calculated over the full 24hrs of day
 mvpathreshold =c(100), # moderate-vig physical activity
 bout.metric = 4,
 excludefirstlast = FALSE,
 includenightcrit = 16,
 #=====================
 # Part 3
 #=====================
 timethreshold=c(5,10),
 anglethreshold=5,
 ignorenonwear= TRUE,

 #=====================
 # Part 4
 #=====================
 def.noc.sleep = 1,
 loglocation= "file path",# this code was removed for the analysis without a diary

relyonguider = FALSE,
 sleeplogidnum = FALSE,
 colid=1,
 coln1=2,
 do.visual = TRUE, #create a visual representation of the overlap between the sleeplog entries and the accelerometer detections
 outliers.only = TRUE, # visualise only for nights with a difference in onset or waking time larger than the variable of argument criterror
 criterror = 4, # minimum number of hours difference between sleep log and accelerometer estimate for the night to be included in the visualisation
 nnights = 8,

 #=====================
 # Part 5
 #=====================
 threshold.lig = c(30), threshold.mod = c(100), threshold.vig = c(400),
 boutcriter = 0.8, boutcriter.in = 0.9, boutcriter.lig = 0.8,
 boutcriter.mvpa = 0.8, boutdur.in = c(1,10,30), boutdur.lig = c(1,10),
 boutdur.mvpa = c(1),
 includedaycrit.part5 = 2/3,
 #=====================
 # Visual report
 #=====================
 timewindow = c("WW"), # Changed to add MM
 viewingwindow=1, # midday is in the centre of plot
 visualreport=TRUE,
 dofirstpage = TRUE,
 do.report=c(2,3,4,5))

| **Table S1: Comparison of socio-demographic characteristics between excluded and included participants** | | | | |
| --- | --- | --- | --- | --- |
|  | | **Inclusion** | |  |
| **Characteristic** | **Overall, N = 1,098^1^** | **Excluded, N = 2631** | **Included, N = 8351** | **p-value^2^** |
| Sex [female] | 636 (58%) | 153 (58%) | 483 (58%) | >0.9 |
| BMI,kg/m^2^ | 28.4±5.7 | 28.9±6.5 | 28.3±5.5 | 0.3 |
| *Unknown* | 58 | 58 | 0 |  |
| Ethnicity |  |  |  | >0.9 |
| *Caucasian* | 1,011 (92%) | 243 (92%) | 768 (92%) |  |
| *Aboriginal* | 5 (0.5%) | 1 (0.4%) | 4 (0.5%) |  |
| *Polynesian* | 6 (0.5%) | 1 (0.4%) | 5 (0.6%) |  |
| *Vietnamese* | 4 (0.4%) | 1 (0.4%) | 3 (0.4%) |  |
| *Chinese* | 30 (2.7%) | 7 (2.7%) | 23 (2.8%) |  |
| *Indian* | 35 (3.2%) | 9 (3.4%) | 26 (3.1%) |  |
| *Other* | 5 (0.5%) | 1 (0.4%) | 4 (0.5%) |  |
| *Unknown* | 3 | 1 | 2 |  |
| Age, years | 56.6±5.7 | 56.3±6.2 | 56.7±5.6 | 0.3 |
| Income |  |  |  | 0.2 |
| *Low* | 297 (29%) | 80 (34%) | 217 (27%) |  |
| *Middle* | 290 (28%) | 61 (26%) | 229 (29%) |  |
| *High* | 444 (43%) | 97 (41%) | 347 (44%) |  |
| *Unknown* | 68 | 26 | 42 |  |
| Education |  |  |  | <0.001 |
| *High school or less* | 259 (25%) | 80 (33%) | 179 (22%) |  |
| *Training after school* | 399 (38%) | 97 (40%) | 302 (38%) |  |
| *University* | 390 (37%) | 68 (28%) | 322 (40%) |  |
| *Unknown* | 51 | 19 | 32 |  |
| Alcohol |  |  |  | 0.7 |
| *Alcohol: Abstainer* | 332 (33%) | 77 (33%) | 255 (33%) |  |
| *Medium consumer* | 515 (51%) | 122 (52%) | 393 (50%) |  |
| *High consumer* | 169 (17%) | 35 (15%) | 134 (17%) |  |
| *Unknown* | 83 | 30 | 53 |  |
| Smoking [yes] | 108 (10%) | 37 (15%) | 71 (8.9%) | 0.003 |
| *Unknown* | 60 | 25 | 35 |  |
| ^1^n (%); Mean±SD | | | | |
| ^2^Pearson's Chi-squared test; Welch Two Sample t-test. | | | | |

| Table S2: Comparison of actigraphy and PSG sleep parameters among participants with moderate-severe OSA (AHI≥15) | | | | | | | | | |
| --- | --- | --- | --- | --- | --- | --- | --- | --- | --- |
| Sleep Parameter | | PSG | Actigraphy | Mean Bias | Absolute Bias | T statistic | Effect size | *p-*value | |
|  |  | Mean ±SD | Mean ±SD | Mean ±SD | Mean ±SD |  |  |  |  |
| *GGIR: Actigraphy* ***with*** *sleep diary (n=258)* | |  |  |  |  |  |  |  | |
| TST, hours | | 5.78±1.06 | 6.35±1.01 | 0.57±1.02 | 0.89±0.75 | 8.92 | 0.56 | *<0.001* | |
| SOL, hours | | 0.31±0.4 | 0.06±0.18 | -0.25±0.42 | 0.29±0.39 | -9.36 | -0.58 | *<0.001* | |
| WASO, hours | | 1.44±0.73 | 1.13±0.76 | -0.31±0.87 | 0.69±0.61 | -5.69 | -0.35 | *<0.001* | |
| SE, % | | 75.11±12.21 | 83.9±11.58 | 8.79±14.02 | 12.8±10.47 | 10.07 | 0.63 | *<0.001* | |
| *GGIR: Actigraphy* ***without*** *sleep diary* (n=257) |  | |  |  |  |  |  |  | |
| TST, hours | | 5.78±1.06 | 6.04±1.47 | 0.25±1.34 | 1.04±0.88 | 3.04 | 0.19 | *<0.001* | |
| WASO, hours | | 1.44±0.73 | 0.93±0.73 | -0.51±0.96 | 0.81±0.73 | -8.59 | -0.54 | *<0.001* | |
| SE, % | | 75.08±12.23 | 86.27±9.86 | 11.19±14.31 | 13.97±11.6 | 12.53 | 0.78 | *<0.001* | |
| *ActiLife: Actigraphy with sleep diary* *(n=257)* | |  |  |  |  |  |  |  | |
| TST, hours | | 5.78±1.06 | 6.71±0.82 | 0.92±0.9 | 1.01±0.81 | 16.40 | 1.13 | *<0.001* | |
| SOL, hours | | 0.31±0.41 | 0.06±0.09 | -0.25±0.42 | 0.26±0.4 | -9.46 | -0.59 | *<0.001* | |
| WASO, hours | | 1.44±0.73 | 0.82±0.55 | -0.62±0.76 | 0.76±0.62 | -13.18 | -0.82 | *<0.001* | |
| SE, % | | 75.09±12.23 | 88.46±7.18 | 13.37±11.78 | 14.05±10.96 | 18.19 | 1.13 | *<0.001* | |
| Comparisons were made using paired t-tests. | | | | | | | | |  |
| Abbreviations: TST = Total sleep time, SOL= Sleep onset latency, WASO = Wake after sleep onset, SE = Sleep efficiency, AHI = apnoea hypopnea index | | | | | | | | |  |
| The sample of actigraphy files assessed in GGIR without a sleep diary was reduced by one participant due to TST being calculated as <2 hours, as per exclusion criteria (n=257). | | | | | | | | |  |
| The sample of actigraphy files processed in ActiLife was reduced by one participant as there was an error with their data being formatted into 60 second epochs (sample, n= 257). | | | | | | | | |  |

| Table S3: Comparison of actigraphy and PSG sleep parameters among participants with no-mild OSA (AHI <15) | | | | | | | | | | |
| --- | --- | --- | --- | --- | --- | --- | --- | --- | --- | --- |
| Sleep Parameter | | PSG | Actigraphy | Mean Bias | Absolute Bias | T statistic | Effect size | *p-*value | | |
|  |  | Mean ±SD | Mean ±SD | Mean ±SD | Mean ±SD |  |  |  |  |  |
| *GGIR: Actigraphy* ***with*** *sleep diary (n=577)* | |  |  |  |  |  |  |  | | |
| TST, hours | | 6.02±0.91 | 6.63±0.73 | 0.61±0.78 | 0.73±0.67 | 18.73 | 0.78 | *<0.001* | | |
| SOL, hours | | 0.29±0.3 | 0.05±0.17 | -0.24±0.31 | 0.27±0.29 | -18.64 | -0.78 | *<0.001* | | |
| WASO, hours | | 1.16±0.68 | 0.79±0.5 | -0.37±0.67 | 0.56±0.52 | -13.24 | -0.55 | *<0.001* | | |
| SE, % | | 79.29±10.95 | 88.69±7.9 | 9.4±11.14 | 10.97±9.59 | 20.27 | 0.84 | *<0.001* | | |
| *GGIR: Actigraphy* ***without*** *sleep diary* (n=575) |  | |  |  |  |  |  |  | | |
| TST, hours | | 6.03±0.89 | 6.56±1.03 | 0.53±1.05 | 0.85±0.81 | 11.98 | 0.50 | *<0.001* | | |
| WASO, hours | | 1.15±0.66 | 0.71±0.41 | -0.44±0.67 | 0.59±0.54 | -15.97 | -0.67 | *<0.001* | | |
| SE, % | | 79.44±10.69 | 89.74±5.81 | 10.3±10.59 | 11.38±9.42 | 23.33 | 0.97 | *<0.001* | | |
| *ActiLife: Actigraphy with sleep diary* *(n=574)* | |  |  |  |  |  |  |  | | |
| TST, hours | | 6.02±0.91 | 6.74±0.73 | 0.72±0.8 | 0.81±0.72 | 21.64 | 1.13 | *<0.001* | | |
| SOL, hours | | 0.29±0.3 | 0.07±0.07 | -0.22±0.3 | 0.24±0.29 | -17.79 | -0.74 | *<0.001* | | |
| WASO, hours | | 1.16±0.68 | 0.69±0.48 | -0.47±0.68 | 0.6±0.56 | -16.76 | -0.70 | *<0.001* | | |
| SE, % | | 79.29±10.98 | 90.03±6.35 | 10.74±10.58 | 11.58±9.65 | 24.33 | 1.02 | *<0.001* | | |
| Comparisons were made using paired t-tests. | | | | | | | | |  |  |
| Abbreviations: TST = Total sleep time, SOL= Sleep onset latency, WASO = Wake after sleep onset, SE = Sleep efficiency, AHI = apnoea hypopnea index | | | | | | | | |  |  |
| The sample of actigraphy files assessed in GGIR without a sleep diary was reduced by three participants due to TST being calculated as <2 hours, as per exclusion criteria (n=575). | | | | | | | | | |  |
| The sample of actigraphy files processed in ActiLife was reduced by three participants as there was an error with their data being formatted into 60 second epochs (sample, n= 574). | | | | | | | | | |  |

Figure S1: Comparison of GGIR actigraphy with a sleep diary and polysomnography (PSG) measurement of SOL on the same night (n=835). A) Correlation of actigraphy and PSG SOL. B) Boxplot (median, first and third quartiles and range) of SOL measured from actigraphy and PSG with paired t-test. C) Bland Altman Plot of actigraphy and PSG SOL: mean difference (^____^), upper and lower limits of agreement (- - -) with 95% confidence interval (shaded) and regression slope (- - -) with 95% confidence interval (shaded). **** p < 0.0001 for significant differences between actigraphy and PSG. Negative SOL values were due to GGIR estimating sleep onset shortly before the diary recorded onset time.


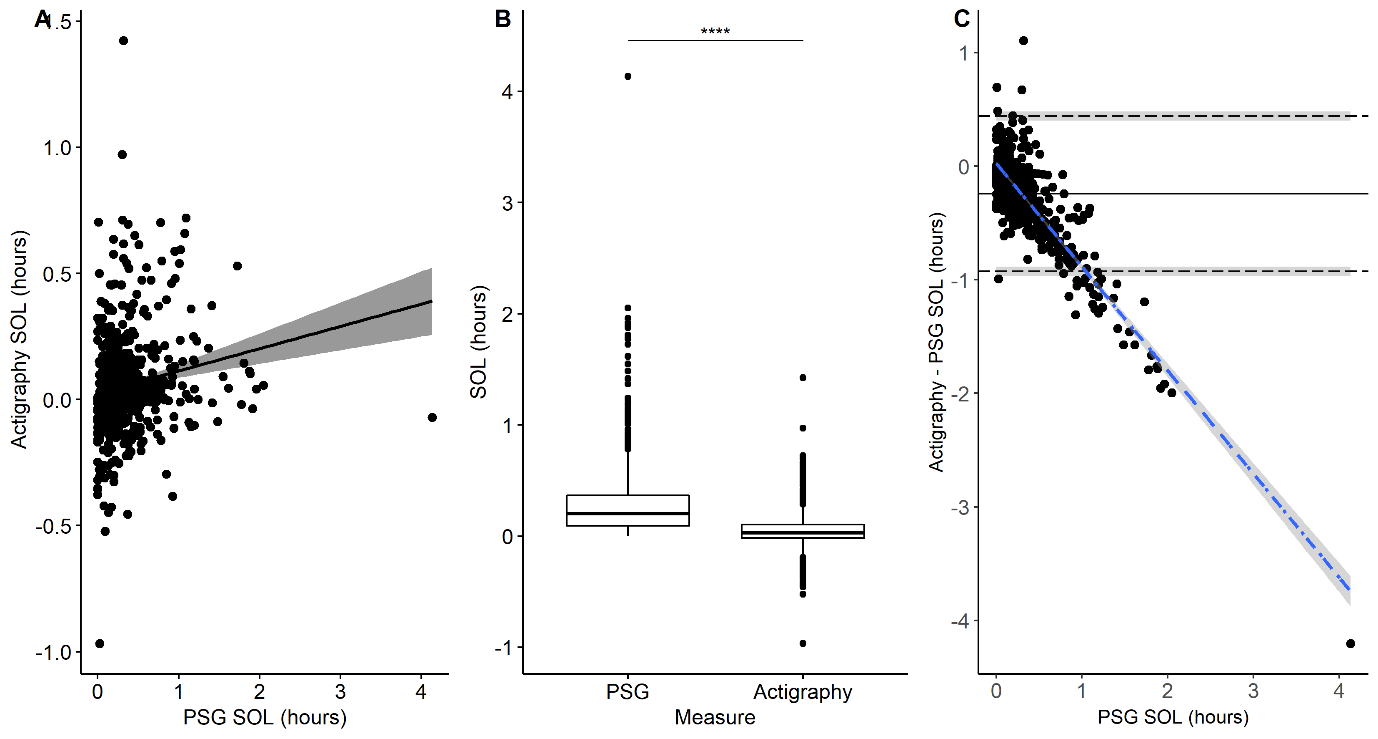


Figure S2: Comparison of GGIR actigraphy with a sleep diary and polysomnography (PSG) measurement of WASO on the same night (n=835). A) Correlation of actigraphy and PSG WASO. B) Boxplot (median, first and third quartiles and range) of WASO measured from actigraphy and PSG with paired t-test. C) Bland Altman Plot of actigraphy and PSG WASO: mean difference (^____^), upper and lower limits of agreement (- - -) with 95% confidence interval (shaded) and regression slope (- - -) with 95% confidence interval (shaded). * p < 0.001 for significant differences between actigraphy and PSG.


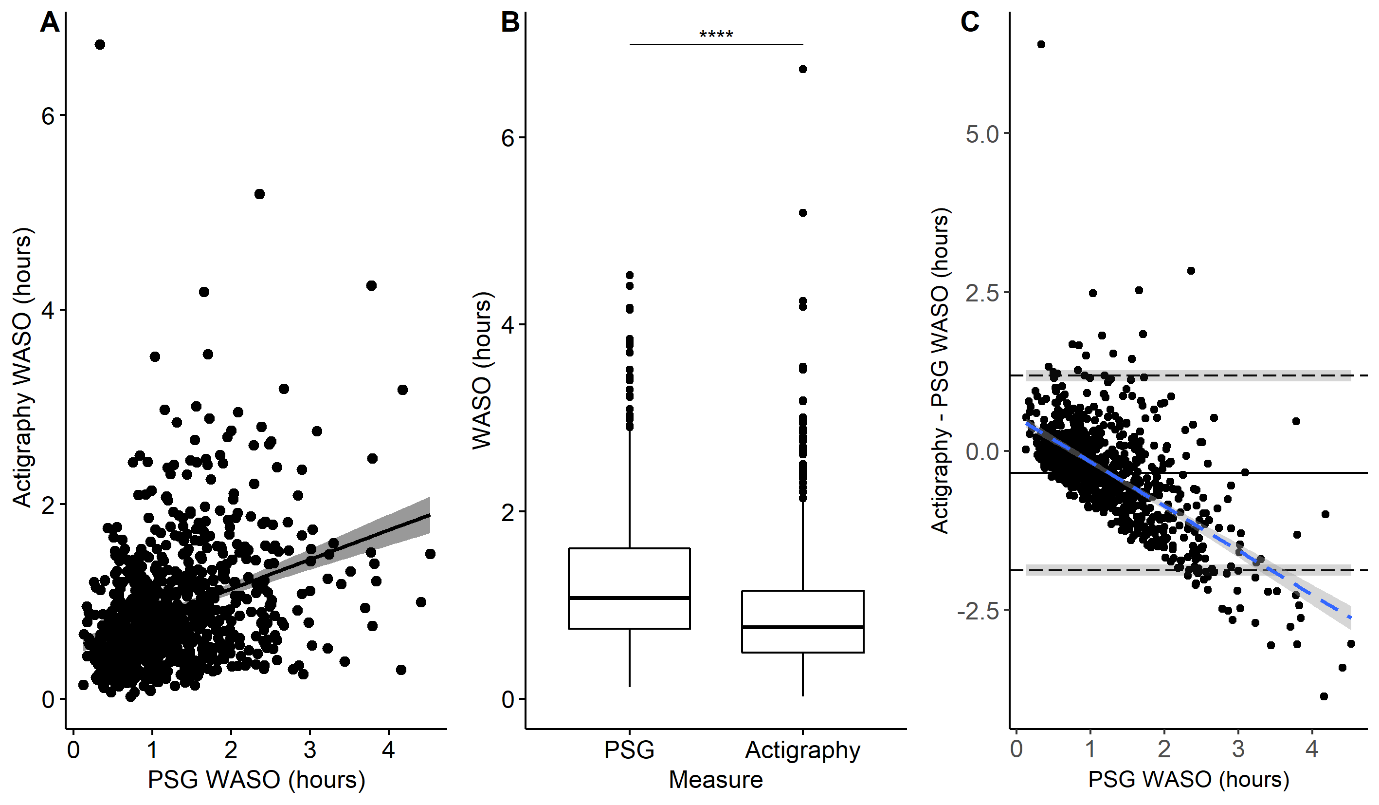


Figure S3: Comparison of GGIR actigraphy with a sleep diary and polysomnography (PSG) measurement of SE on the same night (n=835). A) Correlation of actigraphy and PSG SE. B) Boxplot (median, first and third quartiles and range) of SE measured from actigraphy and PSG with paired t-test. C) Bland Altman Plot of actigraphy and PSG SE: mean difference (^____^), upper and lower limits of agreement (- - -) with 95% confidence interval (shaded) and regression slope (- - -) with 95% confidence interval (shaded). **** p < 0.0001 for significant differences between actigraphy and PSG.


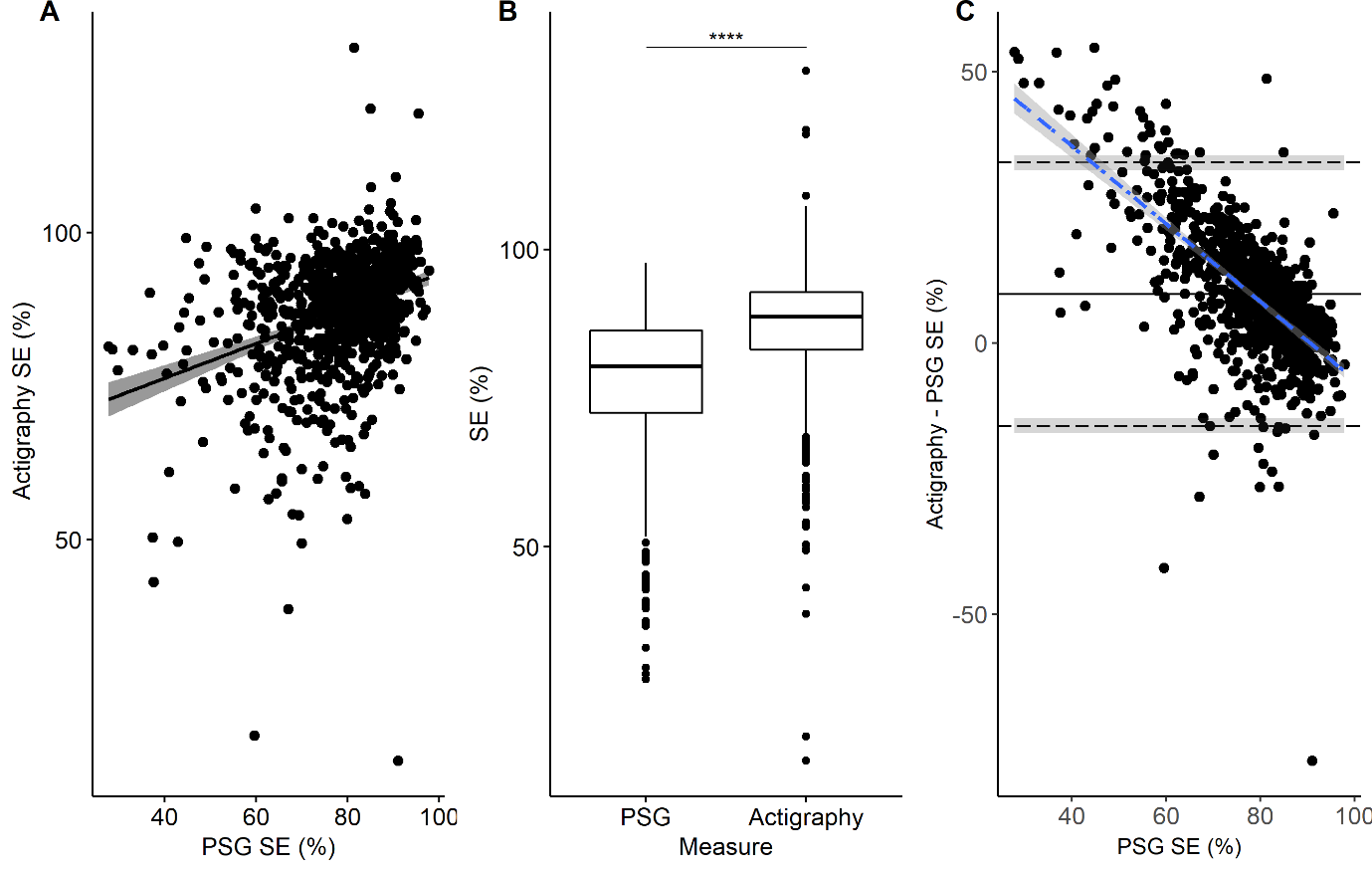


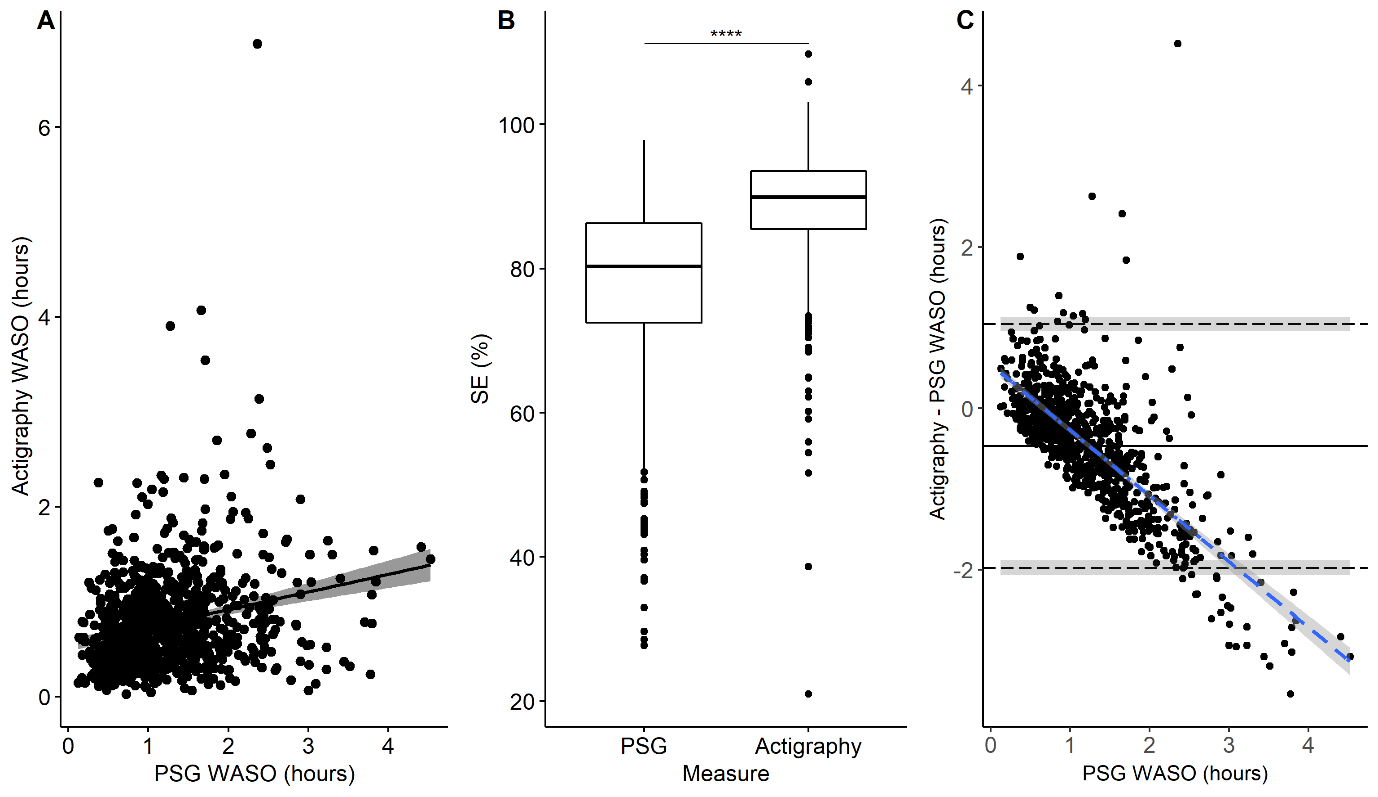


Figure S4: Comparison of GGIR actigraphy without a sleep diary and polysomnography (PSG) measurement of WASO on the same night (n=832). A) Correlation of actigraphy and PSG WASO. B) Boxplot (median, first and third quartiles and range) of WASO measured from actigraphy and PSG with paired t-test. C) Bland Altman Plot of actigraphy and PSG WASO: mean difference (^____^), upper and lower limits of agreement (- - -) with 95% confidence interval (shaded) and regression slope (- - -) with 95% confidence interval (shaded). **** p < 0.0001 for significant differences between actigraphy and PSG.


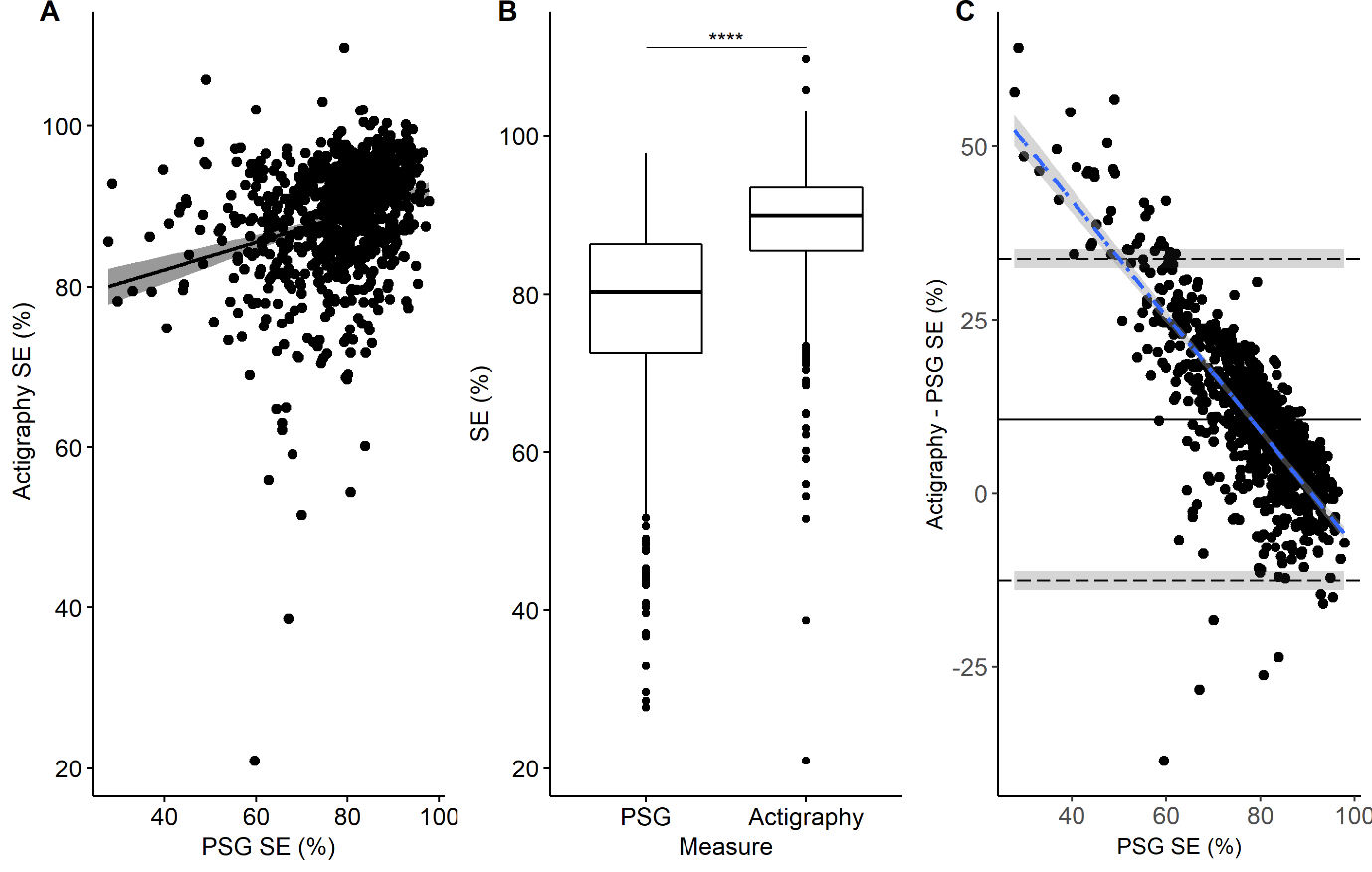


Figure S5: Comparison of GGIR actigraphy without a sleep diary and polysomnography (PSG) measurement of SE on the same night (n=832). A) Correlation of actigraphy and PSG SE. B) Boxplot (median, first and third quartiles and range) of SE measured from actigraphy and PSG with paired t-test. C) Bland Altman Plot of actigraphy and PSG SE: mean difference (^____^), upper and lower limits of agreement (- - -) with 95% confidence interval (shaded) and regression slope (- - -) with 95% confidence interval (shaded). **** p < 0.0001 for significant differences between actigraphy and PSG.


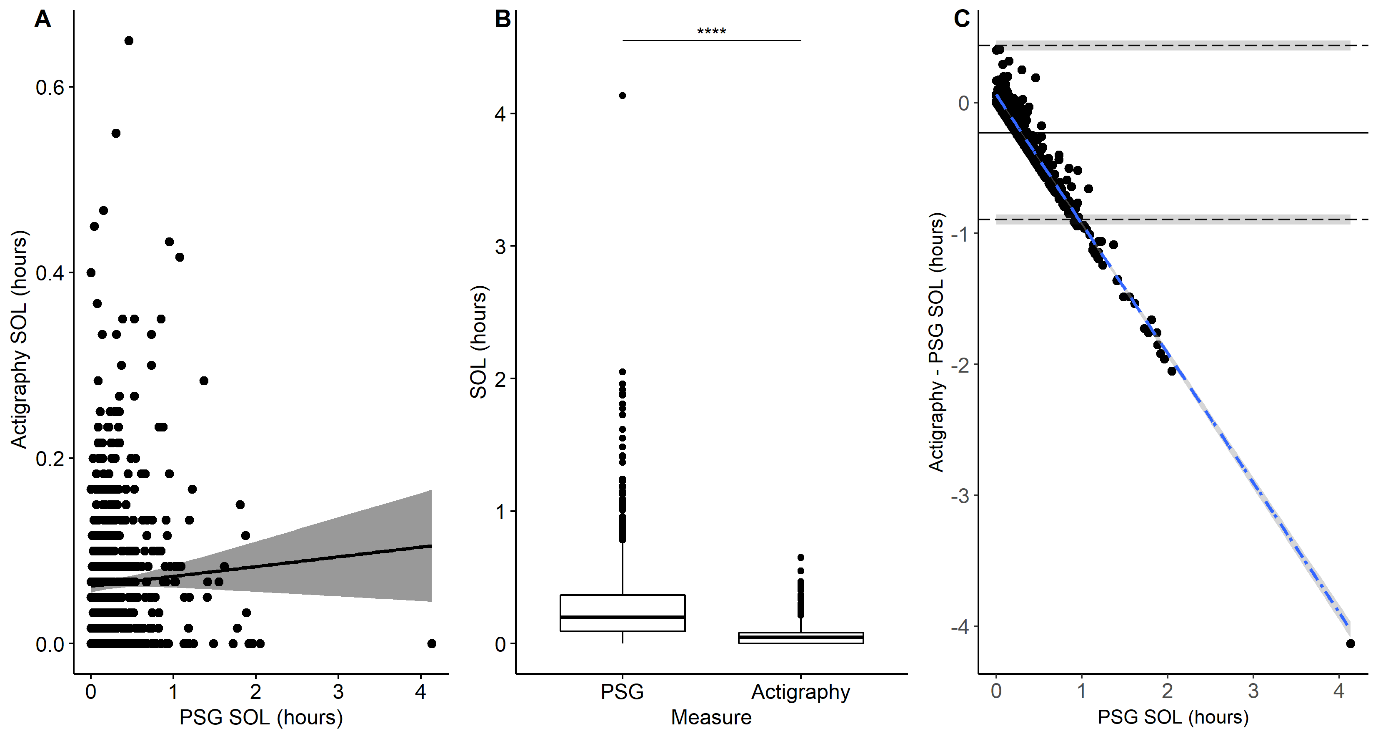


Figure S6: Comparison of ActiLife actigraphy with a sleep diary and polysomnography (PSG) measurement of SOL on the same night (n=831). A) Correlation of actigraphy and PSG SOL. B) Boxplot (median, first and third quartiles and range) of SOL measured from actigraphy and PSG with paired t-test. C) Bland Altman Plot of actigraphy and PSG SOL: mean difference (^____^), upper and lower limits of agreement (- - -) with 95% confidence interval (shaded) and regression slope (- - -) with 95% confidence interval (shaded). **** p < 0.0001 for significant differences between actigraphy and PSG.


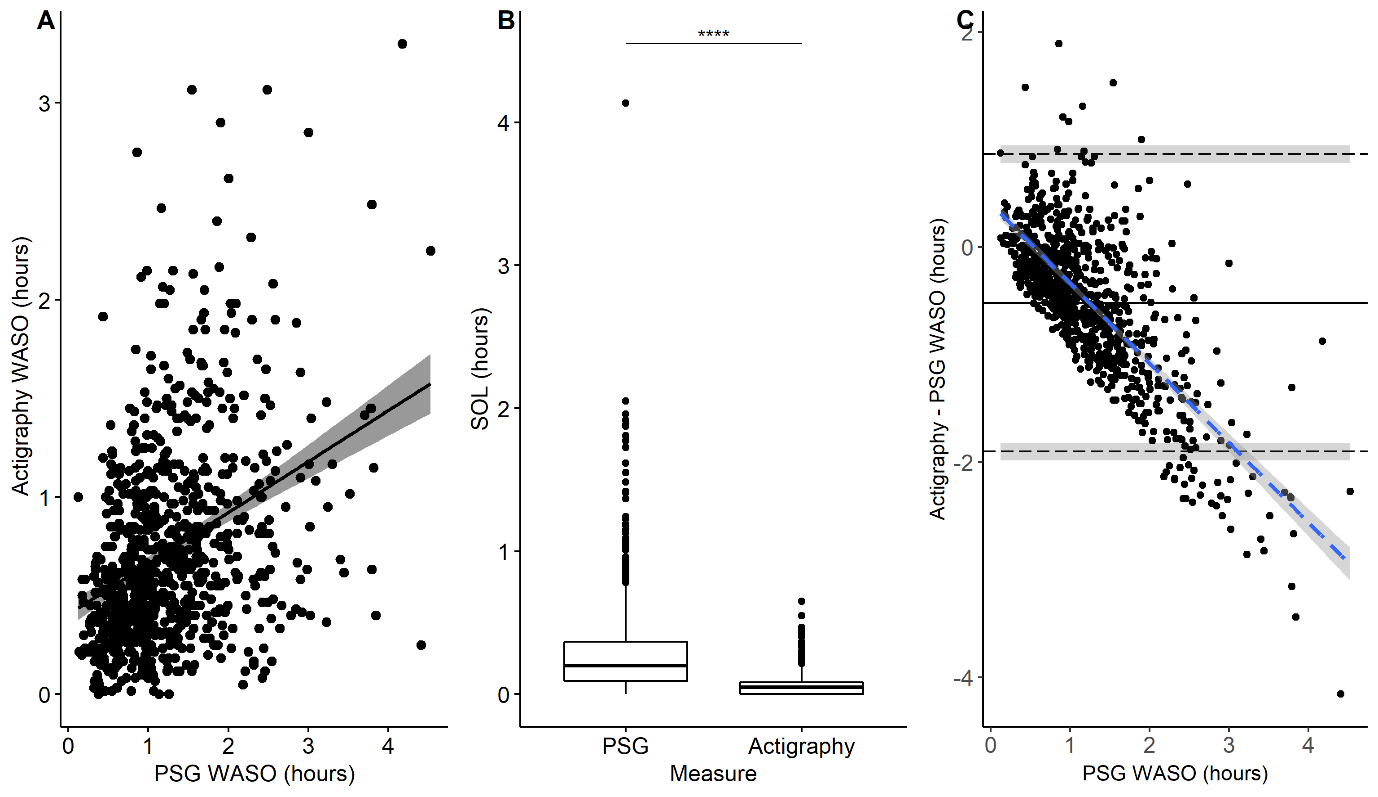


Figure S7: Comparison of ActiLife actigraphy with a sleep diary and polysomnography (PSG) measurement of WASO on the same night (n=831). A) Correlation of actigraphy and PSG WASO. B) Boxplot (median, first and third quartiles and range) of WASO measured from actigraphy and PSG with paired t-test. C) Bland Altman Plot of actigraphy and PSG WASO: mean difference (^____^), upper and lower limits of agreement (- - -) with 95% confidence interval (shaded) and regression slope (- - -) with 95% confidence interval (shaded). * p < 0.001 for significant differences between actigraphy and PSG.


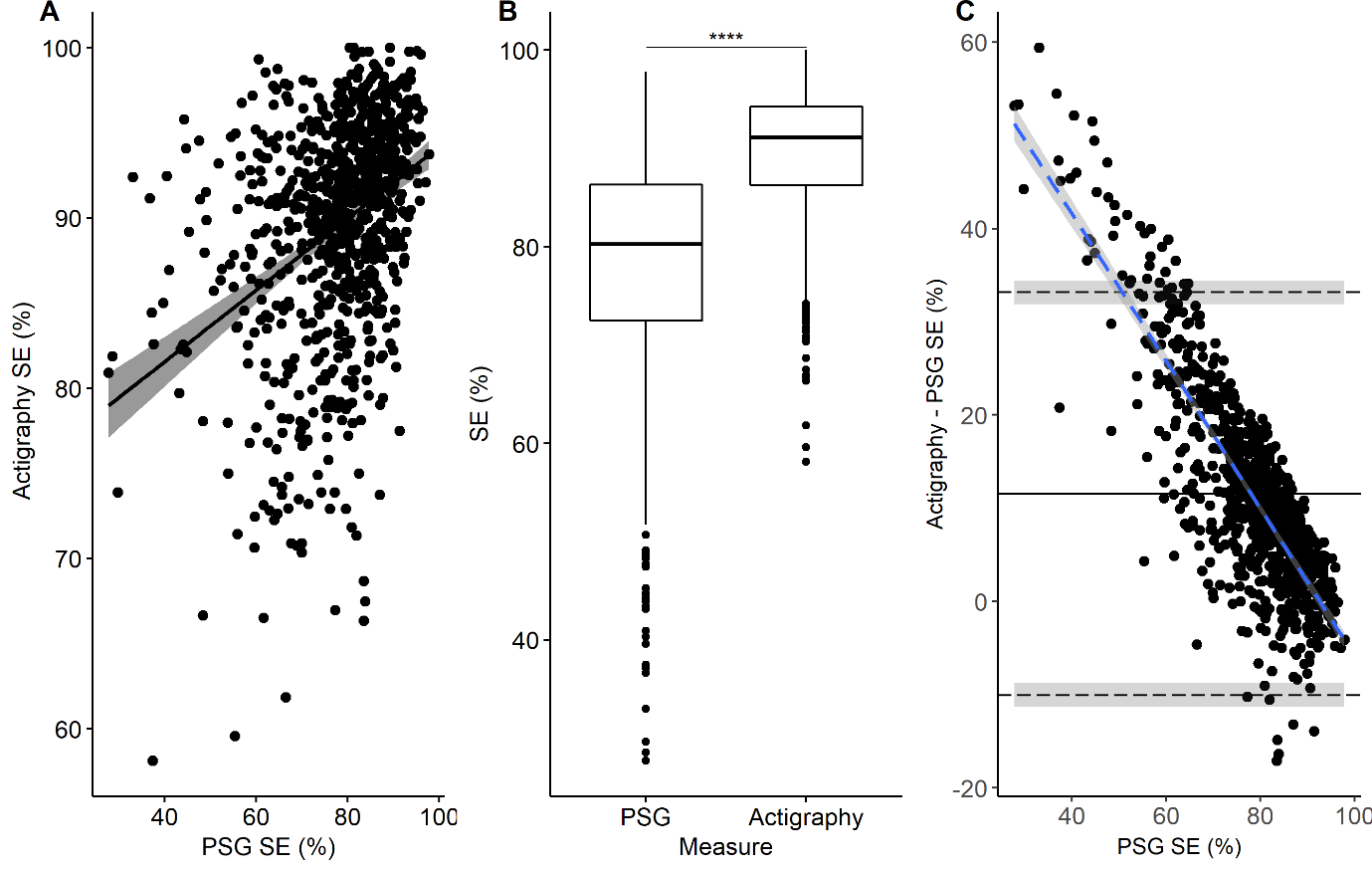


Figure S8: Comparison of ActiLife actigraphy with a sleep diary and polysomnography (PSG) measurement of SE on the same night (n=831). A) Correlation of actigraphy and PSG SE. B) Boxplot (median, first and third quartiles and range) of SE measured from actigraphy and PSG with paired t-test. C) Bland Altman Plot of actigraphy and PSG SE: mean difference (^____^), upper and lower limits of agreement (- - -) with 95% confidence interval (shaded) and regression slope (- - -) with 95% confidence interval (shaded). **** p < 0.0001 for significant differences between actigraphy and PSG.
